# Supplementary material for: Medial packing and elastic asymmetry stabilize the double-gyroid in block copolymers
Source: Nat Commun. 2022 May 12;13:2629. doi: 10.1038/s41467-022-30343-2 (PMC9098509; doi:10.1038/s41467-022-30343-2)
Supplement: Supplementary file 1 — Supplementary Information [file 41467_2022_30343_MOESM1_ESM.pdf]

Supplementary Information: Medial packing and elastic asymmetry stabilize the double-gyroid in block copolymers

Abhiram Reddy, Michael S. Dimitriyev, and Gregory M. Grason
Department of Polymer Science and Engineering,
University of Massachusetts, Amherst, MA 01003

SUPPLEMENTARY NOTE 1. STRONG SEGREGATION THEORY FORMALISM

Here, we review the assumptions and necessary details associated with the strong segregation theory (SST) describing thermodynamic equilibria of self-assembled diblock copolymers [1]. In the $\chi N \to \infty$ limit, chains tend to stretch strongly from the IMDS to reduce the (lateral) area per chain exposure to unlike blocks. Since chain repulsion dominates over configurational entropy in this regime, the dominant configuration of the melt has chain conformations that follow "classical trajectories" [2] which minimize the stretching free energy of chains subject to constraints of incompressibilty in the brush. As a result, blocks of each type are expected follow straight trajectories that extend from the free ends in the bulk of A and B brush to the IMDS, where the junction points are localized. In the $\chi N \to \infty$ limit, the effect of brush interpenetration is subdominant, occurring over a distance that vanishes relative to equilibrium brush height [3]. Hence, capturing the leading free energy terms in SST requires us only to consider brush domains that extend up to the "terminal boundary" that divides the two contacting brushes in a A or B domain [4]. Additionally, in the strong segregation limit (SSL), the width of the interface between A and B blocks vanishes relative to brush height, allowing us to approximate it as a 2D surface to which junction points between A and B are localized.

The *total* free energy $\mathcal{F}$ (in units of $k_{\rm B}T$) of the system of polymer chains in melt has two components

$$\mathcal{F} = \mathcal{F}_{\rm int} + \mathcal{F}_{\rm st} \qquad \text{(Supplementary Equation 1)}$$

where $\mathcal{F}_{\rm int}$ represents the enthalpic contribution due to interblock repulsion at AB interface and $\mathcal{F}_{\rm st} = \mathcal{F}_{\rm st}^{\rm A} + \mathcal{F}_{\rm st}^{\rm B}$ is the entropic cost of chain stretching, decomposed into contributions from A and B molten brush domains [5, 6].

In this study, we compute the stretching energy of chains using two approaches: the *parabolic brush theory* (PBT) [2] and its *end-exclusion zone corrected* (EEZC) form [7, 8]. In the PBT, the free-energy minimizing balance of chain entropy and incompressibilty results in a free energy density that is proportional to the second moment of the volume relative to the IMDS [9]. The stretching free energy contribution from the $\alpha = {\rm A}, {\rm B}$ block has the form

$$\mathcal{F}_{\rm st}^{\alpha} = \frac{3\pi^2 \rho}{8 N_\alpha^2 a_\alpha^2} \int_{V_\alpha} {\rm d}V \ z^2 \qquad \text{(Supplementary Equation 2)}$$

where z is the extension of a chain away from the IMDS along a straight line, as shown in Fig. 2A in the main text. Here, ρ^{-1} is the volume occupied by single monomer unit, N_α is the number of segments in block α and a_α is the corresponding statistical segment length. The volume integral is taken over volume V_α of the brush domains of type α.

The PBT makes implicit assumptions about the free end distribution $g(z)$ throughout incompressible brushes that results in a well-documented error [7] for brushes that splay away from grafting surfaces, usually associated with positive mean curvature of those surfaces, e.g. the outer domains of cylindrical or spherical domains. In those cases, the PBT requires an unphysical negative free end distribution [1], indicating that this theory is strictly only an upper-bound on the true SSL limit for those local regions. The exact SSL requires the introduction of an end-exclusion zone (EEZ) [7], for which $g(z) = 0$, near to the anchoring surface (i.e. the IMDS), and leads to a more complex, self-consistent distribution in the brush, resulting in a free energy density that deviates at least somewhat from the simple $\propto z^2$ from of PBT, i.e. eq. (Supplementary Equation 2). To capture and assess the importance of free energy corrections from the EEZ to the PBT as summarized in Sec. SUPPLEMENTARY NOTE 10, we implement a variant of the approach of ref. [8], described in [10], to capture the exact SSL entropy for molten brush domains of arbitrary local geometries, of the type needed to construct complex DG domains. Notably, for the purposes of comparing the free energies of Lam, Hex and DG near to their phase boundaries, we find that the PBT is sufficient to an excellent approximation. Moreover, given the intuitive geometrical form of the PBT entropy (i.e. second-moment of brush volumes relative to the IMDS) we focus most of the main text presentation on its simpler form, and results derived from it.

To determine the optimal free of a given morphology X, we evaluate the free energy per chain $F(x)$ based on a particular periodic repeat unit of the structure (e.g. a unit cell, or an asymmetric unit), which has a volume $V(x)$, corresponding to $\rho V(X)/N$ chains per unit, where $N = N_{\rm A} + N_{\rm B}$ is the total chain length. Following the definitions

of IMDS area $A(X)$ and second-moments $I_\alpha(X) \equiv \int_{V_\alpha(X)} \mathrm{d}V\, z^2$ in these repeat units and dividing by the number of chains per unit, we have the form of eq. (1) in the main text, where the surface tension of the IMDS is

$$\gamma = \rho \bar{a} \sqrt{\frac{\chi}{6}} \left(\frac{2}{3} \frac{\epsilon_0^{3/2} - \epsilon_0^{-3/2}}{\epsilon_0 - \epsilon_0^{-1}} \right) \quad \text{(Supplementary Equation 3)}$$

where $\bar{a} \equiv \sqrt{a_\mathrm{A} a_\mathrm{B}}$ is the geometric mean of segment lengths and $\epsilon_0 \equiv a_\mathrm{A}/a_\mathrm{B}$ is the conformational asymmetry parameter. The coefficients of the stretching free energies, κ_α, are determined by molecular architecture,

$$\begin{aligned} \kappa_\mathrm{A} &= \frac{3\pi^2 \rho \bar{n}^2}{4N^2 \bar{a}^2} \frac{1}{f^2 \epsilon} \\ \kappa_\mathrm{B} &= \frac{3\pi^2 \rho \bar{n}^2}{4N^2 \bar{a}^2} \frac{\epsilon}{(1-f)^2}, \end{aligned} \quad \text{(Supplementary Equation 4)}$$

where $f = N_\mathrm{A}/N$ is the volume fraction of A-segments per chain and $\epsilon \equiv (n_\mathrm{B} a_\mathrm{A})/(n_\mathrm{A} a_\mathrm{B}) = (n_\mathrm{B}/n_\mathrm{A})\epsilon_0$ is the *elastic asymmetry* parameter defined by Milner for a generalized class of $\mathrm{A}_{n_\mathrm{A}}\mathrm{B}_{n_\mathrm{B}}$ miktoarm star block copolymers [11], and $\bar{n} = \sqrt{n_\mathrm{A} n_\mathrm{B}}$.

Computing $A(X)$ and $I_\alpha(X)$ requires construction of space-filling configurations of local brush regions (with corresponding chain trajectories), divided by an IMDS. For all geometries besides Lam, these local chain geometries are non-uniform, and we approximate the continuous integrals over the brush volume using tessellations into narrow wedge-like volumes that extend from the IMDS in along straight paths up to the terminal boundaries, with the accuracy of the discrete approximation improving with increasingly narrow wedge volumes. In Secs. SUPPLEMENTARY NOTE 3 and SUPPLEMENTARY NOTE 6 we describe the respective approaches to construct tessellations for DG, based on the medial construction, and for Hex, based on a "kinked-path" ansatz.

SUPPLEMENTARY NOTE 2. AREA PER CHAIN AND EQUILIBRIUM THERMODYNAMICS

Here, we describe the SST thermodynamics in terms of the *IMDS area per chain* Σ. Σ varies thermodynamically, with an optimal value that is determined by the competition between IMDS enthalpy and change stretching, which also sets the equilibrium domain size. Additionally, this allows us to compare the free energy of distinct morphologies X by holding them at a constant value Σ, a state at which their free energies derive only from relative differences in stretching energy of block α (either A or B). Equal IMDS area per chain comparisons are shown in main text Figs. 3,4.

Given a system with a reference length scale D_0 (which might correspond e.g. to a the length of translation vector in a periodic structure) and reference mean area per chain Σ_0, the area per chain Σ is related by an appropriate affine rescaling of the system $L_0 \mapsto L = \lambda L_0$. This affine rescaling alters the geometric quantities that enter the free energy as follows

$$\begin{aligned} V_0 &\mapsto V = \lambda^3 V_0 \\ A_0 &\mapsto A = \lambda^2 A_0 \quad , \\ I_{\alpha,0} &\mapsto I_\alpha = \lambda^5 I_{\alpha,0} \end{aligned} \quad \text{(Supplementary Equation 5)}$$

which allows us to express the area per chain Σ using scale factor λ

$$\Sigma = \frac{A_0}{V_0} \frac{N}{\rho} \frac{1}{\lambda} \ . \quad \text{(Supplementary Equation 6)}$$

Alternatively this gives the length scaling needed to set the average area per chain to a given value Σ

$$\lambda = \frac{A_0}{V_0} \frac{N}{\rho} \frac{1}{\Sigma} \ . \quad \text{(Supplementary Equation 7)}$$

From this, we have the free energy of morphology X as a function of area per chain,

$$F(X, \Sigma) = \gamma \Sigma + \frac{N^3}{2\rho^3 \Sigma^2} \left[\kappa_\mathrm{A} \mathcal{S}_\mathrm{A}(X) + \kappa_\mathrm{B} \mathcal{S}_\mathrm{B}(X) \right] \ , \quad \text{(Supplementary Equation 8)}$$

where rescaled dimensionless entropic cost S_α of stretching for each block is defined as

$$\mathcal{S}_\alpha = \frac{I_{\alpha,0}}{V_0} \left(\frac{A_0}{V_0} \right)^2 . \quad \text{(Supplementary Equation 9)}$$

Thus, at fixed mean area per chain Σ, the differences in free energy of competing phases is completely determined by the quantity in the brackets $[\cdots]$ of eq. (Supplementary Equation 8), viz. $S_{\rm A}(X)$ and $S_{\rm B}(X)$ while the coefficients $\kappa_{\rm A}$ and $\kappa_{\rm B}$ are same for all phases at any given molecular parameters.

Finally, we consider the equilibrium domain size by minimizing $F(X, \Sigma)$ with respect to Σ, resulting in a optimal area per chain,

$$\Sigma_*(X) = \frac{N}{\gamma^{1/3}\rho}\big[\kappa_{\rm A}\mathcal{S}_{\rm A}(X) + \kappa_{\rm B}\mathcal{S}_{\rm B}(X)\big]^{1/3} \quad \text{(Supplementary Equation 10)}$$

and equilibrium free energy per chain

$$F_*(X) = \frac{3\gamma^{2/3}N^{1/3}\bar{n}^{2/3}}{2\rho^{2/3}a^{2/3}}\big[\bar{\kappa}_{\rm A}\mathcal{S}_{\rm A}(X) + \bar{\kappa}_{\rm B}\mathcal{S}_{\rm B}(X)\big]^{1/3} \quad \text{(Supplementary Equation 11)}$$

where $\bar{\kappa}_\alpha = N^2\bar{a}^2\rho^{-1}\bar{n}^{-2}\kappa_\alpha$ is the dimensionless block stiffness coefficient, functions of only on f and ϵ

SUPPLEMENTARY NOTE 3. MEDIAL TESSELLATIONS OF TRIPLY-PERIODIC NETWORKS FOR SST

We use the medial map to generate space filling tessellations of triply periodic network morphologies. Here we focus on the example of the DG morphology, but this method generalizes to morphologies of arbitrary shape and topology.

The process begins with a set of orientated generating surfaces $\mathbf{G}$, where the orientation corresponds to directions pointing toward the "inner" and "outer" domains. For the case of the DG network, as we detail below, $\mathbf{G}$ can, for example, be supplied by a level set $F(\mathbf{x}) = 0$ of a function F possessing $I4_132$ symmetry, e.g.

$$F(x,y,z) = \sin\left[\frac{2\pi x}{D}\right]\cos\left[\frac{2\pi y}{D}\right] + \cos\left[\frac{2\pi x}{D}\right]\sin\left[\frac{2\pi z}{D}\right] + \sin\left[\frac{2\pi y}{D}\right]\cos\left[\frac{2\pi z}{D}\right] - \phi_0 \quad \text{(Supplementary Equation 12)}$$

where D is the length of the DG unit cell and ϕ_0 is the variable level set parameter. For $\phi_0 = 0$, this is an approximation of the gyroid G minimal surface. For $\phi_0 \neq 0$, solutions to eq. (Supplementary Equation 12) result in enantiomeric pairs of surfaces (ϕ_0 and $-\phi_0$) that have the same topology as the IMDS separating tubular domains and matrix domain of DG. This level set equation is generalized in a later Sec. SUPPLEMENTARY NOTE 4 below.

Given a set of generating surfaces, we construct a triangular mesh to use as a discrete approximation to these surfaces; to triangulate such implicit surfaces, we use the `CGAL` C++ libraries [12]. We then construct the medial sets of this generating surface $\mathbf{G}$, following a method described in the SI ref. [4]. Briefly, this is done following the algorithm of ref. [13] by finding the Voronoi cells of each vertex of the mesh approximation to $\mathbf{G}$, which is done using the `Voro++` software [14]. The medial sets are then generated by finding the intersection of the normal to the generating surface with the outer boundary of the point's Voronoi cell at its two distant ends (on both sides of the surface), representing locations that are equidistant to multiple points on the boundary mesh. That pair of points are "images" of generating vertex on $\mathbf{G}$ mapped to the medial sets on either side of the generating surface (for DG these correspond to tubular and matrix domains). As a convention for the remainder of this SI, we denote vertices on the medial set of the matrix with a "-" and vertices in the tubular domains with a "+". We denote $\hat{\mathbf{n}}_\pm$ as the medial vector pointing normally from the vertex at $\mathbf{G}$ to its medial image inside the tubular and matrix domains for "+" and "-", respectively. The result is a medial map: for each vertex $\mathbf{v} \in \mathbf{G}$, there is a pair of points $\mathbf{v}_- \in \mathbf{T}_{\rm B}$ (in the matrix domain) and $\mathbf{v}_+ \in \mathbf{T}_{\rm A}$ (in the tubular domain) given by the map $\mathbf{m}_\alpha$,

$$\begin{aligned} \mathbf{m}_\alpha \;:\; & \mathbf{G} \to \mathbf{T}_\alpha \\ & \mathbf{v} \mapsto \mathbf{v}_\alpha = \mathbf{v} + d_\alpha(\mathbf{v})\hat{\mathbf{n}}_\alpha(\mathbf{v}) = \mathbf{m}_\alpha(\mathbf{v}) \end{aligned} \quad \text{(Supplementary Equation 13)}$$

where $d_\alpha(\mathbf{v})$ is the distance between $\mathbf{v}$ and $m_\alpha(\mathbf{v})$ found from the medial set construction. Notice that at this point we identify the medial sets $\mathbf{T}_{\rm A}$ and $\mathbf{T}_{\rm B}$ as *terminal boundaries*, although $\mathbf{G}$ should not be confused with an IMDS as the structure does not necessarily satisfy local balance of volume fractions.

The medial map allows us to decompose space into a set of volumes, each of which is associated to an initial facet, which we label μ, of the initial generating surface mesh. By construction, every mesh facet $(\mathbf{v}_\mu^{(1)}, \mathbf{v}_\mu^{(2)}, \mathbf{v}_\mu^{(3)})$ on $\mathbf{G}$ is mapped to a corresponding facet $(\mathbf{v}_{\alpha,\mu}^{(1)}, \mathbf{v}_{\alpha,\mu}^{(2)}, \mathbf{v}_{\alpha,\mu}^{(3)})$ on $\mathbf{T}_\alpha$, with the connectivity of neighbor edges $\mathbf{T}_\alpha$ inherited from the connectivity of corresponding vertices on $\mathbf{G}$. Since this map occurs via straight lines connecting the vertices of the facets (spanning from "inner" to "outer" medial set), as shown in eq. (Supplementary Equation 13), we can construct a space-filling tessellation of the region between $\mathbf{T}_{\rm A}$ and $\mathbf{T}_{\rm B}$. This polyhedral region spans between associated facets

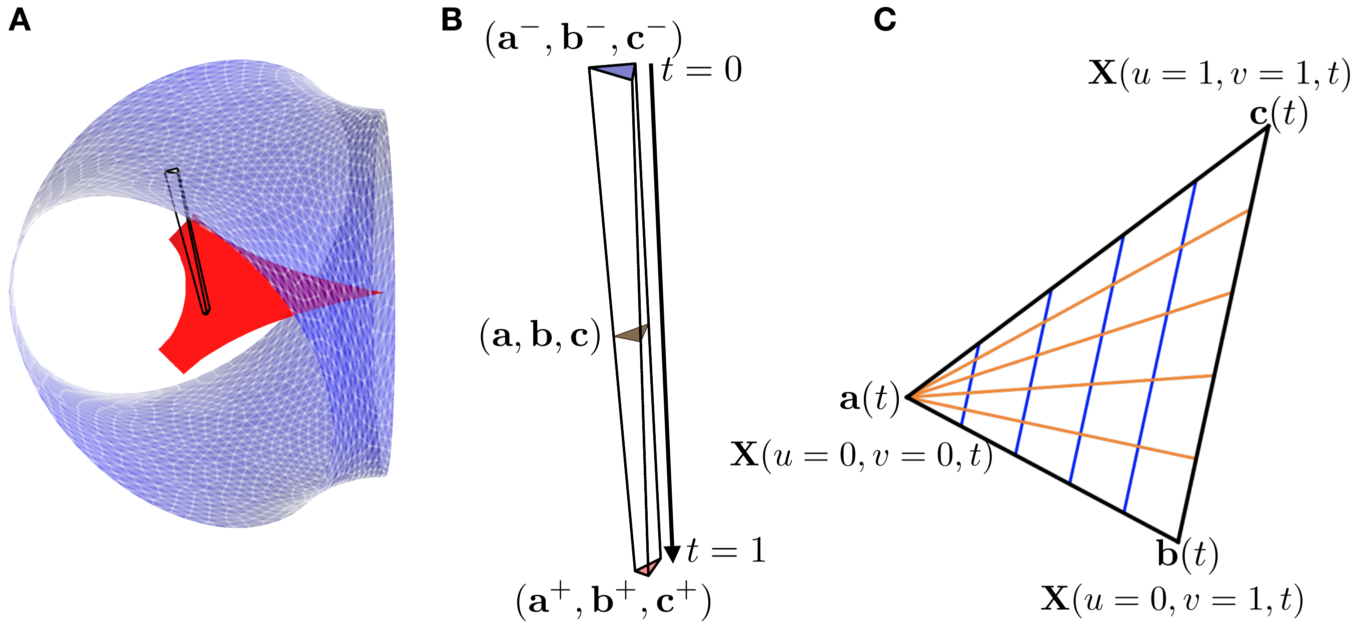

Supplementary Fig. 1. (A) Illustration of the tessellation of a nodal region, bounded by $\mathbf{T}_{\mathrm{A}}$ and $\mathbf{T}_{\mathrm{B}}$, by prismatic wedges. (B) A wedge is capped by faces $(\mathbf{a}^-, \mathbf{b}^-, \mathbf{c}^-)$ on $\mathbf{T}_{\mathrm{B}}$ and $(\mathbf{a}^+, \mathbf{b}^+, \mathbf{c}^+)$ on $\mathbf{T}_{\mathrm{A}}$. Sections of the wedge are parameterized by coordinate $t \in [0, 1]$. (C) Triangular section of the wedge at fixed t, parameterized by coordinates $u, v \in [0, 1]$, with lines of constant u (orange) and v (blue). Any point $\mathbf{X}$ in the wedge can be uniquely specified by (u, v, t).

on these medial sets defined by the points $\{\mathbf{v}_{\mathrm{A}}^{(1)}, \mathbf{v}_{\mathrm{A}}^{(2)}, \mathbf{v}_{\mathrm{A}}^{(3)}\}$ in $\mathbf{T}_{\mathrm{A}}$, forming one triangular face and $\{\mathbf{v}_{\mathrm{B}}^{(1)}, \mathbf{v}_{\mathrm{B}}^{(2)}, \mathbf{v}_{\mathrm{B}}^{(3)}\}$ in $\mathbf{T}_{\mathrm{B}}$, forming a second triangular face. Pairs of identified vertices $\{(\mathbf{v}_{\mathrm{A}}^{(1)}, \mathbf{v}_{\mathrm{B}}^{(1)}), (\mathbf{v}_{\mathrm{A}}^{(2)}, \mathbf{v}_{\mathrm{B}}^{(2)}), (\mathbf{v}_{\mathrm{A}}^{(3)}, \mathbf{v}_{\mathrm{B}}^{(3)})\}$ form three edges connecting the triangular faces, adding three quadrilateral faces to the resulting polyhedron, resulting in a 5-face polyhedron, or pentahedron. Note that the quadrilateral faces may be non-planar in general, so the resulting shape is a curved pentahedron, which we shall refer to as a "wedge." Next we describe a simple map that spans the interior of these wedges such that quadrilateral faces of the wedges are ruled surfaces that perfectly match without gaps from wedge to neighbor wedge, and therefore the collection of triangular prismatic wedges on either side of $\mathbf{G}$ is space filling between medial sets $\mathbf{T}_{\mathrm{A}}$ and $\mathbf{T}_{\mathrm{B}}$.

We parameterize the interior of volume-tessellating wedges by considering a mapping between 2D triangular sections of the wedges – parameterized by coordinates (u, v) – and a coordinate t that translates along the length of the wedge to all points $\mathbf{X}$ within the wedge interior. To construct the coordinates we consider a given wedge with associated connected triangular facets on $\mathbf{T}_{\mathrm{B}}$ and $\mathbf{T}_{\mathrm{A}}$, which we denote as the "-" and "+" faces of the wedge. The vertices on those respective faces are denoted by $\{\mathbf{a}_-, \mathbf{b}_-, \mathbf{c}_-\}$ and $\{\mathbf{a}_+, \mathbf{b}_+, \mathbf{c}_+\}$ such that each pair of vertices $\mathbf{a}_\pm$, $\mathbf{b}_\pm$, and $\mathbf{c}_\pm$ are associated by the medial map of the generating surface. The edge vectors connecting $\mathbf{T}_{\mathrm{B}}$ to $\mathbf{T}_{\mathrm{A}}$ are given by

$$\begin{aligned} \mathbf{C}_a &= \mathbf{a}_+ - \mathbf{a}_- \\ \mathbf{C}_b &= \mathbf{b}_+ - \mathbf{b}_- \\ \mathbf{C}_c &= \mathbf{c}_+ - \mathbf{c}_- \end{aligned} \qquad \text{(Supplementary Equation 14)}$$

where $\mathbf{C}_a = \mathbf{a}_+ - \mathbf{a}_-$, $\mathbf{C}_b = \mathbf{b}_+ - \mathbf{b}_-$, and $\mathbf{C}_c = \mathbf{c}_+ - \mathbf{c}_-$. Given these edge vectors, we define a linear map along the three edges $\mathbf{a}(t)$, $\mathbf{b}(t)$, and $\mathbf{c}(t)$

$$\begin{aligned} \mathbf{a}(t) &= \mathbf{a}_- + \mathbf{C}_a t \\ \mathbf{b}(t) &= \mathbf{b}_- + \mathbf{C}_b t \\ \mathbf{c}(t) &= \mathbf{c}_- + \mathbf{C}_c t \end{aligned} \qquad \text{(Supplementary Equation 15)}$$

where $t \in [0, 1]$ denotes the length fraction along each edge such that $\mathbf{a}(0) = \mathbf{a}_-$, $\mathbf{a}(1) = \mathbf{a}_+$, $\mathbf{b}(0) = \mathbf{b}_-$, $\mathbf{b}(1) = \mathbf{b}_+$, $\mathbf{c}(0) = \mathbf{c}_-$, and $\mathbf{c}(1) = \mathbf{c}_+$. At given t, these three vertices define a triangular region — defined by $\{\mathbf{a}(t), \mathbf{b}(t), \mathbf{c}(t)\}$ — that continuously interpolates between the upper facet on $\mathbf{T}_{\mathrm{B}}$ at $t = 0$ and bottom facet on $\mathbf{T}_{\mathrm{A}}$ at $t = 1$. Taking advantage of the convexity of each triangular region at fixed t, any point $\mathbf{X}$ within a triangle can be described by a

pair of coordinates $(u,v) \in [0,1]\times[0,1]$, via

$$\mathbf{X}(u,v,t) = (1-v)\mathbf{a}(t) + v(1-u)\mathbf{b}(t) + uv\mathbf{c}(t) \qquad \text{(Supplementary Equation 16)}$$

giving us a coordinate system (u,v,t) for any point within a wedge (i.e. sweeping each coordinate from 0 to 1 spans the full volume interior to the wedge). Careful consideration shows that quadrilateral (long) faces of wedges are ruled surfaces, where the edge vectors at constant t are the rulings. Moreover, these rulings are identical for face sharing neighbor wedges, since the linear map with t from - to + vertices is the same for both wedges. Hence, and crucially, this ensures that wedge faces match perfectly without gaps.

Using eq. (Supplementary Equation 16), the differential volume element is given by

$$\mathrm{d}V = \mathrm{d}u\ \mathrm{d}v\ \mathrm{d}t \left[\frac{\partial \mathbf{X}}{\partial t}\cdot\left(\frac{\partial \mathbf{X}}{\partial u}\times\frac{\partial \mathbf{X}}{\partial v}\right)\right] \qquad \text{(Supplementary Equation 17)}$$

where the $J(u,v,t) = \left[\frac{\partial \mathbf{X}}{\partial t}\cdot\left(\frac{\partial \mathbf{X}}{\partial u}\times\frac{\partial \mathbf{X}}{\partial v}\right)\right]$ is the determinant of the Jacobian of the coordinate mapping $(u,v,t)\mapsto \mathbf{X}$. To evaluate the Jacobian, we note that the partial derivatives of $\mathbf{X}$ are given by

$$\begin{aligned}\frac{\partial \mathbf{X}}{\partial u} &= v\mathbf{e}_{bc}(t)\\ \frac{\partial \mathbf{X}}{\partial v} &= \mathbf{e}_{ba}(t) + v\mathbf{e}_{bc}(t)\\ \frac{\partial \mathbf{X}}{\partial t} &= (1-v)\mathbf{C}_a + v(1-u)\mathbf{C}_b + uv\mathbf{C}_c\end{aligned} \qquad \text{(Supplementary Equation 18)}$$

where $\mathbf{e}_{ba}(t) \equiv \mathbf{a}(t) - \mathbf{b}(t)$ and $\mathbf{e}_{bc}(t) \equiv \mathbf{c}(t) - \mathbf{b}(t)$ are the edge vectors of the triangle formed at fixed t. Note that map from (u,v,t) to $\mathbf{X}$ is one-to-one for all points in the wedge provided that $J(u,v,t)$ is positive definite, which corresponds to the condition that the interior facet area is non-vanishing at all $t\in[0,1]$. Integrating over cross-section coordinates u and v, we find an expression for the amount of volume $\mathrm{d}V(t)$ per edge fraction interval $\mathrm{d}t$, given by

$$\begin{aligned}\frac{\partial V(t)}{\partial t} &= \iint_0^1 \mathrm{d}u\,\mathrm{d}v\ \left[\frac{\partial \mathbf{X}}{\partial t}\cdot\left(\frac{\partial \mathbf{X}}{\partial u}\times\frac{\partial \mathbf{X}}{\partial v}\right)\right]\\ &= \frac{\mathbf{C}}{2}\cdot[\mathbf{e}_{bc}(t)\times\mathbf{e}_{ba}(t)]\end{aligned} \qquad \text{(Supplementary Equation 19)}$$

where the centroidal vector $\mathbf{C} \equiv (\mathbf{C}_a + \mathbf{C}_b + \mathbf{C}_c)/3$ joins the centroid of triangle facet at $\mathbf{T}_\mathrm{A}$ to the centroid of the facet at $\mathbf{T}_\mathrm{B}$. The length of $\mathbf{C}$ provides a measure of wedge height, so we define $h \equiv |\mathbf{C}|$. Expanding $\partial V/\partial t$ in powers of t, we find

$$\frac{\partial V(t)}{\partial t} = \mathbf{C}\cdot\mathbf{A}_-\left[1 + \frac{\mathbf{C}\cdot(\boldsymbol{\beta}_1+\boldsymbol{\beta}_2)}{2\mathbf{C}\cdot\mathbf{A}_-}t + \frac{\mathbf{C}\cdot\boldsymbol{\gamma}}{2\mathbf{C}\cdot\mathbf{A}_-}t^2\right], \qquad \text{(Supplementary Equation 20)}$$

where $\mathbf{A}_- \equiv \mathbf{e}_{bc}(0)\times\mathbf{e}_{ba}(0)/2$ is the oriented area of the triangular facet at $t=0$, and $\boldsymbol{\beta}_1$, $\boldsymbol{\beta}_2$ and $\boldsymbol{\gamma}$ are vectors describing the splay geometry of the three connecting edges and are given by

$$\begin{aligned}\boldsymbol{\beta}_1 &\equiv (\mathbf{C}_c - \mathbf{C}_b)\times\mathbf{e}_{ba}(0)\\ \boldsymbol{\beta}_2 &\equiv \mathbf{e}_{bc}(0)\times(\mathbf{C}_a - \mathbf{C}_b)\\ \boldsymbol{\gamma} &\equiv (\mathbf{C}_c\times\mathbf{C}_a) + (\mathbf{C}_b\times\mathbf{C}_c) + (\mathbf{C}_a\times\mathbf{C}_b)\end{aligned} \qquad \text{(Supplementary Equation 21)}$$

The splay of the three edge vectors controls the rate of change of volume with the parameter t in a manner that captures the equivalent Steiner's law describing the area $A_\mu(z)$ of surfaces parallel to a curved surface at $z=0$ in terms of mean curvature H and Gaussian curvature K_G via $A(z) = A(0)(1+2Hz+K_Gz^2)$ [15]. Identifying each power of t with the appropriate dimensionless "wedge curvatures," we have the wedge version of Steiner's law,

$$A_\mu(z) = \frac{\partial V}{\partial z} = \hat{\mathbf{C}}\cdot\mathbf{A}_-(1+2\tilde{H}z+\tilde{K}z^2), \qquad \text{(Supplementary Equation 22)}$$

where height along the wedge (measured from $\mathbf{T}_\mathrm{A}$) can be defined as $z\equiv ht$ and the wedge analogues of mean and Gaussian curvatures for the wedge are given by

$$\begin{aligned}\tilde{H} &\equiv \frac{\mathbf{C}\cdot(\boldsymbol{\beta}_1+\boldsymbol{\beta}_2)}{4h\mathbf{C}\cdot\mathbf{A}_-}\\ \tilde{K} &\equiv \frac{\mathbf{C}\cdot\boldsymbol{\gamma}}{2h^2\mathbf{C}\cdot\mathbf{A}_-}\end{aligned} \qquad \text{(Supplementary Equation 23)}$$

respectively. Here we use the notation $\tilde{H}$ and $\tilde{K}$ to clarify that these do not correspond the curvatures of the IMDS, or the terminal boundaries, as the wedges are in general tilted with respect to the normals of those surfaces

Given this area distribution, it is straightforward to compute the volume of wedge within height h,

$$V_\mu(z) = \int_0^z \mathrm{d}y\, A_\mu(y) = \hat{\mathbf{C}} \cdot \mathbf{A}_- z \left(1 + \tilde{H}z + \frac{1}{3}\tilde{K}z^2\right) \quad \text{(Supplementary Equation 24)}$$

On either side of the IMDS, the wedges enclose polymer blocks with junctions bound to a common point, leading to the condition of *volume balance* in each wedge. Specifically, the region spanning from the IMDS to $\mathbf{T}_\mathrm{B}$ must enclose a fraction $1 - f$ of the total volume of the wedge (and the corresponding fraction on the A-side encloses f). With this in mind, we determine the balanced height h_b of the IMDS within each wedge according to the condition

$$V_\mu(h_\mathrm{b}) = (1 - f)V_\mu(h). \quad \text{(Supplementary Equation 25)}$$

where $z = h$ is the total wedge height. Defining the height fraction of the dividing interface $t_\mathrm{b} = h_\mathrm{b}/h$, the volume balance condition is determined by the solution of a cubic equation,

$$t_\mathrm{b}^3 \frac{\tilde{K}h^2}{3} + t_\mathrm{b}^2 \tilde{H}h + t_\mathrm{b} - (1 - f)(1 + \tilde{K}h^2/3 + \tilde{H}h) = 0. \quad \text{(Supplementary Equation 26)}$$

We solve this cubic equation for the real root $0 < t_\mathrm{b} < 1$ in each wedge to set the location of the intersection of the IMDS with the wedges.

Given this balanced IMDS location in each wedge it is straightforward to compute the second moments of volume relative to the IMDS for each wedge μ,

$$I_{\mu,\mathrm{A}} = \int_{h_\mathrm{b}}^{h} \mathrm{d}z\, \hat{\mathbf{C}} \cdot \mathbf{A}_- \big(1 + 2\tilde{H}z + \tilde{K}z^2\big)\big(z - h_\mathrm{b}\big)^2 = \frac{\hat{\mathbf{C}} \cdot \mathbf{A}_-(h - h_\mathrm{b})^3}{3}\Big[1 + \frac{\tilde{H}}{2}(h_\mathrm{b} + 3h) + \frac{\tilde{K}}{10}(h_\mathrm{b}^2 + 3h_\mathrm{b}h + 6h^2)\Big]$$
(Supplementary Equation 27)

and

$$I_{\mu,\mathrm{B}} = \int_0^{h_\mathrm{b}} \mathrm{d}z\, \hat{\mathbf{C}} \cdot \mathbf{A}_- \big(1 + 2\tilde{H}z + \tilde{K}z^2\big)\big(h_\mathrm{b} - z\big)^2 = \frac{\hat{\mathbf{C}} \cdot \mathbf{A}_-}{3}\Big[h_\mathrm{b}^3 + \frac{1}{2}\tilde{H}h_\mathrm{b}^4 + \frac{1}{10}\tilde{K}h_\mathrm{b}^5\Big]. \quad \text{(Supplementary Equation 28)}$$

The total stretching moments per cell computed via simple sum over the wedges within the repeat unit volume

$$I_\alpha = \sum_{\mu \in V_0} I_{\mu,\alpha}. \quad \text{(Supplementary Equation 29)}$$

Solving for the dividing plane in each of the wedges ensures that volumes of A and B blocks occupy volume on either side of IMDS at the proper ratio. However, since the positions of the dividing planes are determined for each wedge independently, the surface formed from the collection of dividing planes is generally not continuous, as depicted in Fig. 2. To determine a reasonably smooth model of the IMDS we determine the location of the IMDS $\mathbf{v}_{i,\mathrm{I}}$ along the edge vector that connects $\mathbf{v}_{i,\mathrm{A}}$ to $\mathbf{v}_{i,\mathrm{B}}$ as follows. Denote the neighbor set of wedges μ (corresponding to facets) that share the vertex i as $\mathcal{N}_i$. The fractional distance of the IMDS is simply the average of the vertex positions for the volume balance facets from wedges that share that common vertex. That is,

$$\mathbf{v}_{i,\mathrm{I}} = \mathbf{v}_{i,\mathrm{B}} + \langle t_i \rangle \big(\mathbf{v}_{i,\mathrm{A}} - \mathbf{v}_{i,\mathrm{B}}\big), \quad \text{(Supplementary Equation 30)}$$

where

$$\langle t_i \rangle = \frac{1}{|\mathcal{N}_i|} \sum_{\mu \in \mathcal{N}_i} t_\mathrm{b}(\mu) \quad \text{(Supplementary Equation 31)}$$

where $t_\mathrm{b}(\mu)$ is balanced fractional height in wedge μ. From this construction, the IMDS is approximated by a triangular mesh whose positions closely follow the locations of balanced facets within the wedge, but with different orientations (see Fig. 3) below. This construction only *slightly* alters the local volume balance in exchange for ensuring a continuous mesh approximation to the IMDS.

Given the area elements of this IMDS mesh $A_{\mu,\mathrm{I}}$, the IMDS area used in the medial SST calculations follows from simple sum within the repeat volume

$$A_0 = \sum_{\mu \in V_0} A_{\mu,\mathrm{I}}. \quad \text{(Supplementary Equation 32)}$$

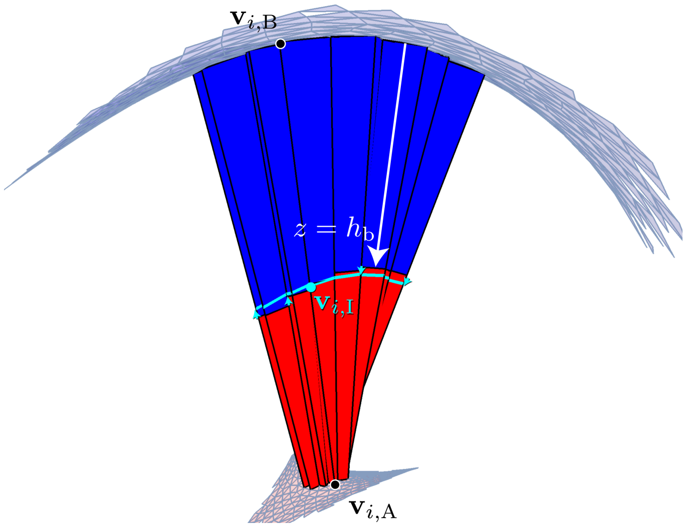

Supplementary Fig. 2. Schematic illustrating the joining of balanced wedges to form a continuous, balanced IMDS. The fraction of red to blue in each wedge is determined by f, with a dividing plane at $z = h_\mathrm{b}$. The dividing planes are then joined together such that neighboring facets meet at vertices $\mathbf{v}_{i,\mathrm{I}}$ to form a continuous IMDS (cyan).

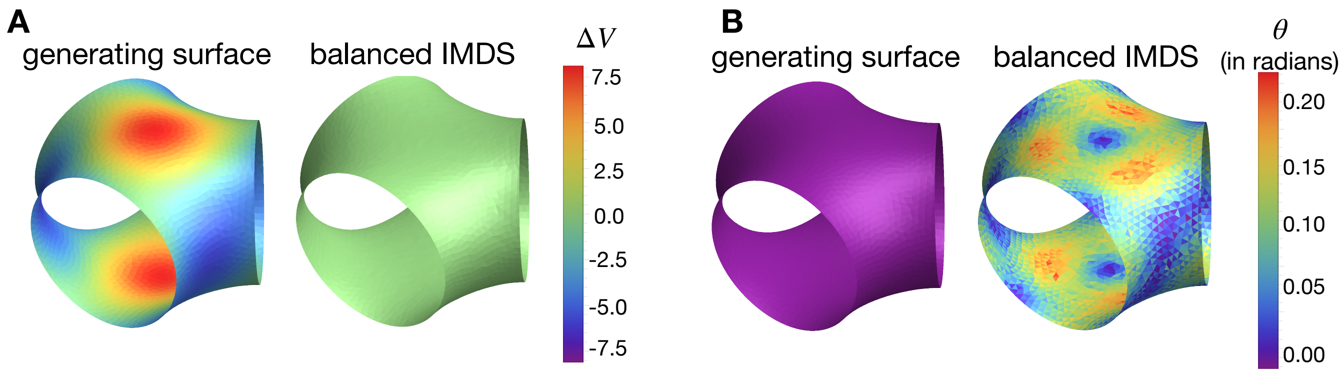

Supplementary Fig. 3. (A) Comparison of deviation from local volume balance for a generating surface (left) and a balanced IMDS (right). While the generating surface may balance the total volume, only the balanced IMDS achieves local balance. (B) Comparison of tilt between surface normals and wedge orientation for a generating surface (left) and a balanced IMDS (right). The medial property ensures that wedges are aligned with the normal of the generating surfaces, which cannot be maintained if the local volume balance constraint is enforced.

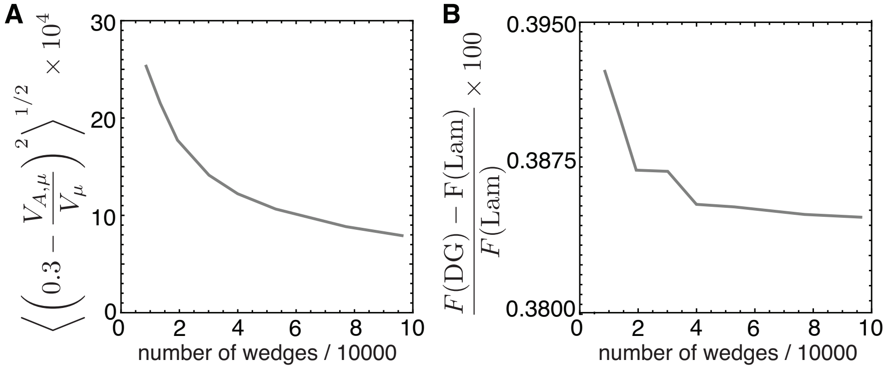

Supplementary Fig. 4. (A) Variance of deviation of local volume balance for fixed $\epsilon = 1$, $f = 0.3$ upon IMDS adjustment is plotted for varying number of wedges per tessellation and in (B) we plot the relative change to $F(\mathrm{DG})$ for the same conditions as in (A).

In Fig. 3 we illustrate the consequences of volume balance, which leads to differences between the generating surface $\mathbf{G}$ and the final IMDS (shown respectively on the left and right of panels in the figure). The figure shows a generating surface (a level set model of a tubular DG surface) whose combined interiors encloses 50% of the total volume. Targeting a composition $f = 0.5$, this surface is balanced globally (i.e. the tubular and matrix domains each enclose 50% of the volume) but not locally. This is shown my mapping the local differences in volume elements $\delta V_{\mathrm{A/B}}$ in the wedges on either side of the IMDS, via the quantity

$$\Delta V = \frac{\delta V_{\mathrm{A}} - \delta V_{\mathrm{B}}}{\delta V_{\mathrm{A}} + \delta V_{\mathrm{B}}}, \qquad \text{(Supplementary Equation 33)}$$

which is plotted in Fig. 3A. Viewed as two molten brushed sharing a common anchoring surface at the IMDS, the local density of A chains reaching the IMDS at any point must equal to the number reaching it from the B side, since they share a common junction. Hence, locally unbalanced configurations (i.e. where $\Delta V \neq 0$) do not correspond to space-filling configurations of AB diblocks, which is the case for the initial generating surface. More generally, for $f \neq 0$, the local balance condition corresponds to $\delta V_{\mathrm{A}}/\delta V_{\mathrm{B}} = f/(1-f)$ everywhere.

Performing the volume balance operation as described above results in a uniform ΔV, but as alluded to above leads to a reorientation of the IMDS relative to the orientation of the wedges. This can be seen by comparing the maps of local tilt shown in Fig. 3B for the unbalanced generating surface to the balanced IMDS. The tilt angle θ is measured by the relative orientation between the generating surface or IMDS normal $\hat{\mathbf{N}}$ and the wedge vector $\mathbf{C}$ that describes the mean trajectory of the chains in the wedge

$$\theta = \arccos\left(\hat{\mathbf{N}} \cdot \mathbf{C}/h\right). \qquad \text{(Supplementary Equation 34)}$$

Fig. 3B shows that the wedges are normal to the generating surface, which follows directly from the medial construction that maps points in the domain onto the closest points on $\mathbf{G}$. In contrast, trajectories in balanced DG are variably tilted away from the IMDS normal. Hence, we may view the tilt of chains relative to the IMDS as measure of *deviation* of the packing from a strictly medial geometry. Below, in Sec. SUPPLEMENTARY NOTE 8 we revisit this morphological feature in the comparison between medial SST and SCF predictions of DG structure.

Our medial constructions ultimately derive from the finite triangulations of the generating surface, i.e. the number of wedges in the final structure is equal to the number of generating surface facets in the repeat volume. To minimize the resolution dependence of our medial SST construction we consider wedge elements within a single *nodal unit* of DG, a 3-valent region as shown in Fig. 1 B-C of the main text, which encloses one of the 16b Wyckoff positions of $Ia\bar{3}d$. Our final calculations derive from tessellations that include roughly 60,000 wedges per nodal unit. To assess the error introduced by this discretization, in Fig. 4A we plot the variance of the deviation of the local volume balance for a fixed $f = 0.30$ upon increasing the number of wedges per single nodal unit and show that the error is small and decreases with finer tessellation, which shows that the IMDS adjustment leads to corrections of order less than 10^{-3} of the number of wedges used. We also show the changes to the free energy per chain within a single nodal region for the same $f = 0.30$ by plotting the percentage change relative to Lam in Fig. 4B. Beyond the 60,000 number of wedges per tessellation, the changes in fractional free energy are $\lesssim 10^{-3}\%$. As we show in Sec. SUPPLEMENTARY NOTE 10 where we consider explicitly the corrections of the end-exclusion zone correction, free energy corrections at this scale (less than the corrections due to end-exclusion) have a negligible effect on predicted phase boundaries.

SUPPLEMENTARY NOTE 4. PARAMETRIC VARIATION OF GENERATING SURFACES

Here we describe the variational framework we use to compute the thermodynamic optimal packing of block copolymers according to medial SST by sampling over a range of terminal geometries for given network symmetry. In this paper, the focus is on DG structures, but the methodology generalizes to other symmetries. The approach is to minimize the SST free energy based on balanced medial packings sampling over broad class of generating surfaces $\mathbf{G}$ whose symmetries are consistent with the $Ia\bar{3}d$ symmetry of DG morphologies, but differ in shape. For convenience, we parameterize the variable $\mathbf{G}$ as level sets the space group $I4_132$, which is a subgroup of $Ia\bar{3}d$. Specifically, level sets of this lower symmetry are used to generate one enantiomeric tubular single-gyroid (SG) surface, which we then invert and superpose to generate the DG structure. We represent these surfaces as the level set $F(x, y, z) = 0$ with Fourier series representation

$$F(x, y, z) = \phi_1 F_{1,1,0}(x, y, z) + \phi_2 F_{2,1,1}(x, y, z) + \phi_3 F_{2,2,0}(x, y, z) + ... - \phi_0 \qquad \text{(Supplementary Equation 35)}$$

where basis functions $F_{hkl}(x, y, z)$ are collections of Fourier modes in the (hkl) Bragg plane, where the possible (hkl) planes are determined by the symmetries of the single gyroid $I4_132$, ϕ_i are (real) coefficients of the basis functions,

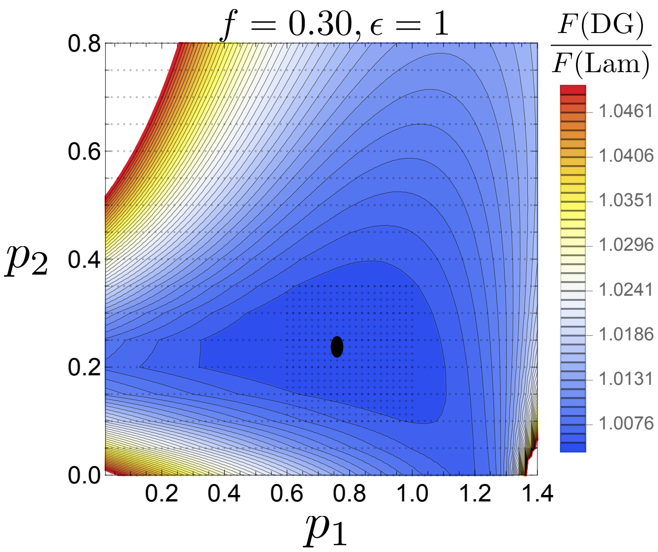

Supplementary Fig. 5. Interpolated free energy landscape describing the results of the medial SST construction with $f = 30$ and $\epsilon = 1$. Each small point drawn over the contour plot represents a single generating surface, parameterized by p_1 and p_2. To better resolve the location of the free energy minimum, a finer grid of generating surfaces was sampled near the expected free energy minimum (large point).

and ϕ_0 is a parameter that shifts the level sets (or equivalently the (000) mode of the Fourier expansion). Note that the ordering of ϕ_i in relation to indices (hkl) is determined by the magnitude of the Bragg vector, $\sqrt{h^2+k^2+l^2}$.

For our calculations, it is sufficient to set the periodicity of the reference unit cell to $D_0 = 1$. In this study, we consider the set of single-gyroid surfaces within the first 3 Fourier modes, the level sets of which are parameterized by $\phi_0/\phi_1 = p_1$ and $\phi_2/\phi_1 = p_2$ which constitute the 2D variational space of generating surface shapes. These first two basis functions are

$$F_{1,1,0}(x,y,z) = \sin(2\pi x)\cos(2\pi y) + \cos(2\pi x)\sin(2\pi z) + \sin(2\pi y)\cos(2\pi z) \qquad \text{(Supplementary Equation 36)}$$

and

$$\begin{aligned} F_{2,1,1}(x,y,z) = \sin(4\pi x)\cos(2\pi y)\sin(2\pi z) + \sin(4\pi y)\cos(2\pi z)\sin(2\pi x) \\ + \sin(4\pi z)\cos(2\pi x)\sin(2\pi y) \end{aligned} \qquad \text{(Supplementary Equation 37)}$$

A DG structure of interwoven single gyroids is constructed from the pair of positive and negative level set parameters $\pm\phi_0$ for given set of ϕ_1 and ϕ_2 (i.e. $p_i \mapsto -p_i$). Notably, volume fractions enclosed in these generating surfaces are *not constrained* to be close to ultimate target volume fraction of the medial SST morphology.

To systematically apply the medial SST procedure established in the previous sections (i.e. find the medial sets, determine the balanced wedge tessellation, and finally compute the free energy), we discretize the space of generating surfaces $\mathbf{G}$ into a 2D grid of p_1 and p_2 parameters. We vary p_1 in intervals of 0.02 and for p_2 we start with intervals of 0.05. As described in Sec. SUPPLEMENTARY NOTE 3, the medial sets of a given $\mathbf{G}$ are used to generate terminal boundaries of the tubular and matrix domains, $\mathbf{T}_\mathrm{A}$ and $\mathbf{T}_\mathrm{B}$, from which we can build a space-filling and locally volume-balanced tessellation by wedges. Using the IMDS areas and second-moments of volume of these wedges and eq. (Supplementary Equation 11), we compute the free energy of each structure over a discrete 2D parameter grid for a given pair of fixed molecular parameters (ϵ, f), leading to a free energy density landscape as shown in Fig. 5. We ensure that the grid is over a sufficient data range that we can bracket a free energy minimum for a fixed (f, ϵ). In the vicinity of the minimum of free energy, we further refine the grid by adding grid points in p_2 with an interval of 0.0125 to determine the minimum free energy via an interpolation in Mathematica. We find that this procedure ensures that for range of f near the $F(\mathrm{Lam}) = \mathrm{F(Hex)}$ phase boundary for a particular ϵ, the minimum values of (p_1, p_2) smoothly varies with f. We illustrate this in Fig. 5, where we show the medial SST free energy landscape for DG morphology at a fixed set of molecular details, $f = 0.3$ and $\epsilon = 1$ and also the grid points in (p_1, p_2) where the data was sampled. By varying the terminal boundaries and associated tessellations of DG, we arrive at thermodynamically optimal packing of chains in strong segregation limit. We summarize the steps involved in medial SST as described above in SUPPLEMENTARY NOTE 3-SUPPLEMENTARY NOTE 4 as a flowchart in Fig. 7

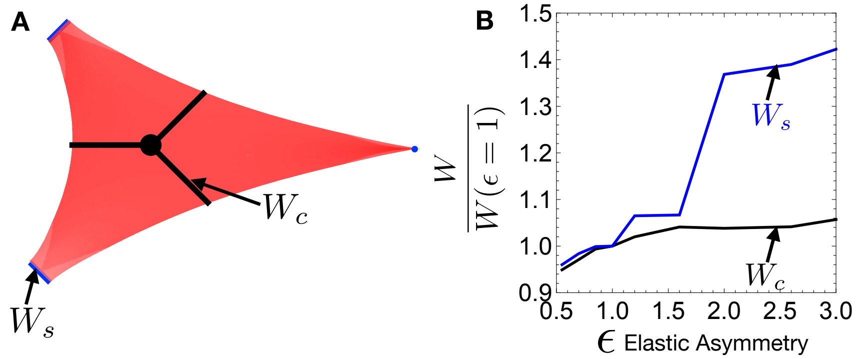

Supplementary Fig. 6. Changing dimensions of the medial web as a function of ϵ. (A) The two metrics of medial web shape we consider are the centroidal width W_c (distance between the centroid and the nearest boundary of the medial web) and the strut width W_s (minimal distance between the two boundary segments along the strut joining neighboring nodes). (B) Both widths, normalized by their $\epsilon = 1$ values, plotted as a function of ϵ. Both dimensions increase with ϵ, but the strut width W_s shows a remarkable transition as $\epsilon \to 2$, indicating a change in the organization of chains in the tubular domain.

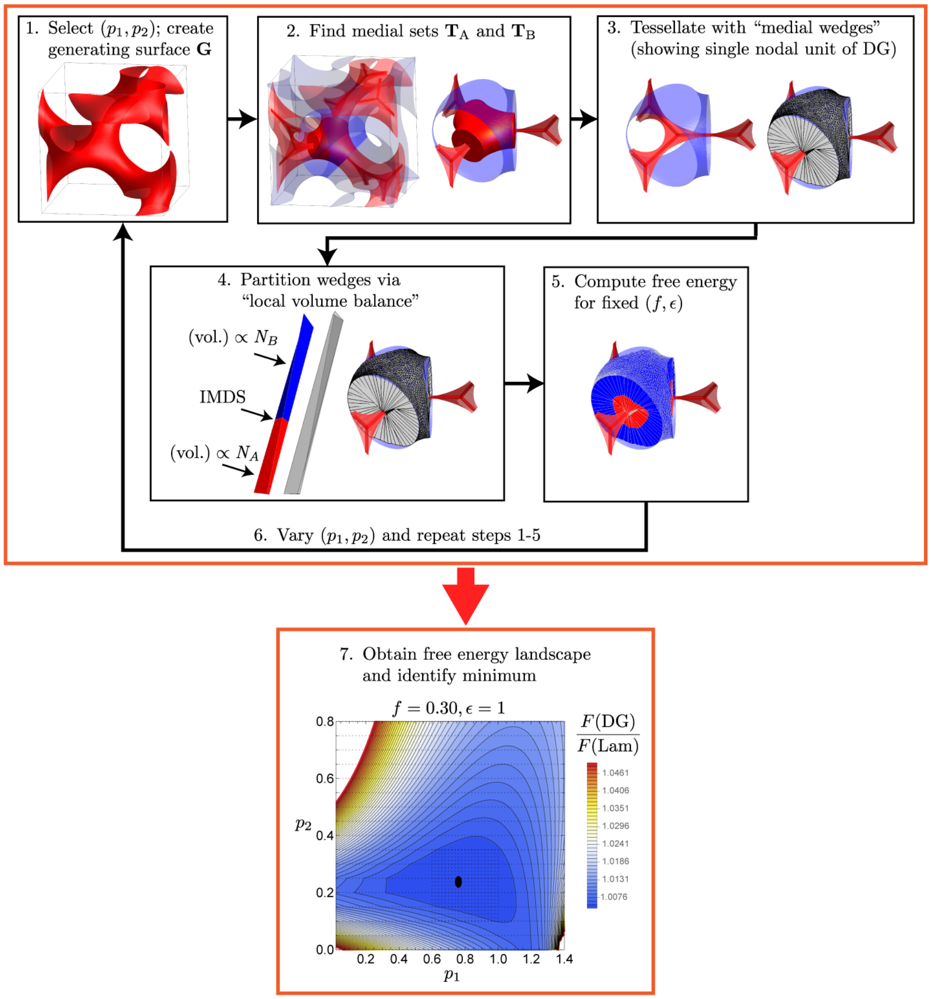

Supplementary Fig. 7. Workflow illustrating our approach to medial strong segregation theory for computing thermodynamically optimal packing of chains in double-gyroid.

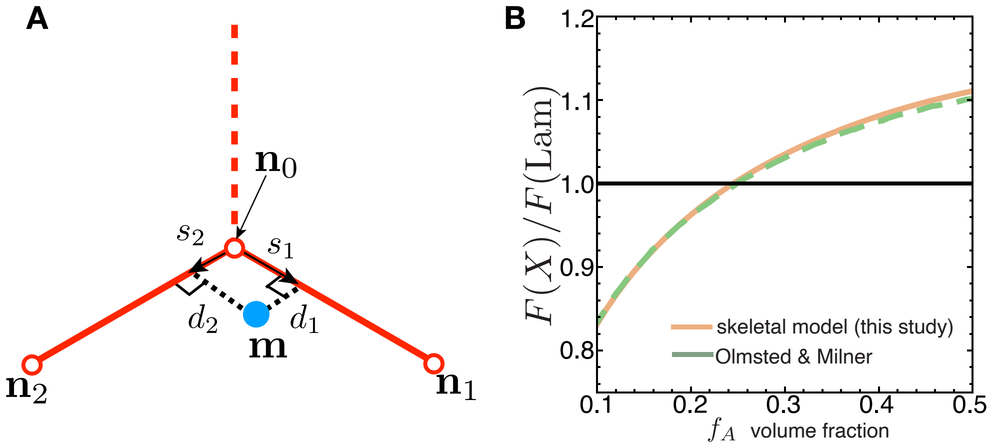

Supplementary Fig. 8. (A) Schematic illustrating our procedure to compute skeletal map for each vertex $\mathbf{m}$ on discrete mesh of tubular medial set. (B) Free energy per chain comparison of our skeletal SST calculation for $\epsilon = 1$ with that of Olmsted and Milner [5]

SUPPLEMENTARY NOTE 5. SKELETAL ANSATZ STRONG SEGREGATION THEORY

Here, we outline our approach to constructing a *skeletal ansatz* for SST calculations of DG. In particular, we aim to closely recapitulate the prior SST approach of Olmsted-Milner (OM) [5], which via a different method, constructed tessellating wedges that span from vertices on the G minimal surface (i.e. the terminal boundary in the matrix domain) to the 1D skeletal graphs, which is taken as the terminal boundary of the tubular domains. The OM approach considers a subsequent free energy optimization of the distribution of anchoring points on the skeleton.

Here, we construct an analog to the OM skeletal structure which is generated from medial tessellations. In short, the process has three steps: First, generate the medial maps ($\mathbf{T}_{\rm A}$ and $\mathbf{T}_{\rm B}$) from given generating surface $\mathbf{G}$. Second, map the terminal points $\mathbf{T}_{\rm A}$ onto the skeletal graph that spans the 16b Wyckoff positions of $Ia\bar{3}d$ (it can be shown that these graphs always lie in the medial set of the tubular domains). Last, balance the wedges and construct the IMDS for area and second-moment calculations.

The first and last steps of the skeletal SST follow the identical approach used for the medial packings. Here, we describe the additional algorithm used to map vertices in $\mathbf{T}_{\rm A}$ to the skeleton $\mathbf{S}$. This operation is performed independently for each vertex on $\mathbf{T}_{\rm A}$, transferring the facet connectivity from the original $\mathbf{G}$ to the final skeletal map, in effect "folding" triangular elements onto the 1D graph. Our skeletal map proceeds as follows. Consider a vertex $\mathbf{m}$ on $\mathbf{T}_{\rm A}$. Identify the three closest nodes in the skeletal graph, and denote these as $\mathbf{n}_0$, $\mathbf{n}_1$ and $\mathbf{n}_2$, indexed in order of increasing distance. This set of nodes corresponds to two adjacent edges of the graph $\mathbf{n}_0 \to \mathbf{n}_1$ and $\mathbf{n}_0 \to \mathbf{n}_2$ (see Fig. 8A). By definition, $\mathbf{m}$ is closer to $\mathbf{n}_0 \to \mathbf{n}_1$ than $\mathbf{n}_0 \to \mathbf{n}_2$.

Naively, we'd expect an optimal skeletal packing to minimally displace $\mathbf{m}$ to $\mathbf{S}$, that is, map onto the closest point on $\mathbf{S}$. However, such a map leads to "jumps" in the map for $\mathbf{m}$ close to $\mathbf{n}_0$ where the closest distance jumps discontinuously from one edge to the next for two nearby points on $\mathbf{T}_{\rm A}$. This discontinuity, in turn, leads to gaps and seams in the resulting volume balanced IMDS, resulting in free energies that far exceed the upper bounds of the OM skeletal ansatz.

To achieve a map from $\mathbf{T}_{\rm A}$ to the skeleton $\mathbf{S}$ that is smooth in the neighborhood of the junction points (corresponding to wedges located in the "elbow" region of DG), we use a map that interpolates between the two closest locations on the two closest edges for a given point. In practice we do this by parameterizing the spans between $\mathbf{n}_0 \to \mathbf{n}_m$ (where $m = 1, 2$) as

$$\mathbf{e}_m(s_m) = \mathbf{n}_0 + s_m(\mathbf{n}_m - \mathbf{n}_0), \qquad \text{(Supplementary Equation 38)}$$

where $s_m \in [0, 1]$ is a parameter that spans the edge. The point of closest contact s_m^* along the m edge is

$$s_m^* = \begin{cases} 0 & \text{for } (\mathbf{m}-\mathbf{n}_0)\cdot(\mathbf{n}_m-\mathbf{n}_0) < 0 \\ \frac{(\mathbf{m}-\mathbf{n}_0)\cdot(\mathbf{n}_m-\mathbf{n}_0)}{|\mathbf{n}_m-\mathbf{n}_0|^2} & \text{for } 0 \leq (\mathbf{m}-\mathbf{n}_0)\cdot(\mathbf{n}_m-\mathbf{n}_0) \leq |\mathbf{n}_m-\mathbf{n}_0|^2 \\ 1 & \text{for } (\mathbf{m}-\mathbf{n}_0)\cdot(\mathbf{n}_m-\mathbf{n}_0) \leq |\mathbf{n}_m-\mathbf{n}_0|^2 > 1 \end{cases}, \qquad \text{(Supplementary Equation 39)}$$

and the corresponding closest distance to those edges is $d_m = |\mathbf{m} - \mathbf{e}_m(s_m^*)|$. Based on these distances, our map associates $\mathbf{m}$ to the region spanned by the two nearest edges by constructing a composite coordinate σ such that

$S = s_1$ for $S \geq 0$ (and maps along e_1) and $S = -s_2$ for $S < 0$ (and maps along $\mathbf{e}_2$). In this way, we determine the mapped location of $\mathbf{m}$ along this span $\sigma(\mathbf{m})$ by a weighted average of the closest points

$$\sigma(\mathbf{m}) = \frac{s_1 w(d_1) - s_2 w(d_2)}{w(d_1) + w(d_2)}, \qquad \text{(Supplementary Equation 40)}$$

where $w(d) = d^{-q}$ are weighting functions of distance. It is straightforward to show that $S(\mathbf{m}) \geq 0$ by construction, and the skeletal map is always along the closest edge (unless at the node $\mathbf{n}_0$ itself), so that the map to $\mathbf{S}$ is given by

$$\mathbf{S}(\mathbf{m}) = \mathbf{e}_1\big(\sigma(\mathbf{m})\big). \qquad \text{(Supplementary Equation 41)}$$

To further optimise the distribution of mapped points onto $\mathbf{S}$ we consider a range of the exponents $q = 1, 2, 3$, and select the value (along with the optimal generating surface) that minimizes the SST free energy of skeletal DG for a given set of (f, ϵ). In our study, we find that $q = 2$ solutions have lowest energy compared to the other exponents and we compare the free energy per chain in Fig. 8B with that of Olmsted & Milner's skeletal model, which we read off Fig. 16 of ref. [5]. At $\epsilon = 1, f = 0.3$, $F(\text{DG})$ from the skeletal model studied in this work is 0.4% higher than Olmsted & Milner's result. In comparison with $F(\text{DG})$ at the same f from medial SST, the relative difference in free energy between medial and skeletal SST is 3.1%.

SUPPLEMENTARY NOTE 6. SST MODEL OF HEX PHASE

To compute the SST free energy of the competitor Hex phase we follow the method of ref. [16]. Here, we consider a model where columnar domains are arranged in a hexagonal lattice and the terminal boundaries of the outer matrix phase are composed of the hexagonal Voronoi cells, while we assume that the the inner terminal boundary of the core block is a 1D line in the center of the column. It is well established that the lattice packing of the columnar phase introduces at least some measure of packing frustration due to fact that rays extending from the center of the column extend variable distances to reach the hexagonal outer terminal boundary. Depending on the relative distance of the IMDS from the outer terminal boundary as well as the ratio of the coronal to core stiffness (parameterized by ϵ), this can lead to warping of the IMDS shape away from the constant curvature cylindrical shape.

To account for this, the SST ansatz of ref. [16] considers a variable class of IMDS shapes, controlled by a variational parameter $\alpha \in [0, 1]$, that interpolates continuously between a circular cross section ($\alpha = 0$) to a perfect hexagonal cross section ($\alpha = 1$) at fixed enclosed core area fraction. While the inner (A) blocks extend radially from the cell center, volume balance and the variable coronal thickness requires the outer blocks to tilt relative to the radial direction, by some degree that varies with position and interface shape (degree of tilting increases for round IMDS shapes that approach the outer terminal boundary and vanishes for IMDS shapes that are affine copies of the hexagonal cell shape).

The variational calculation for Hex minimizes the SST free energy over the IMDS shape, providing an upper bound (within the PBT approximation of brush entropy) of the equilibrium free energy. In contrast to the tessellation of DG into a finite (but very large) number of wedge volumes, the free energy in this "kinked path" ansatz for Hex can be evaluated without explicit discretization of the geometry, by numerical integration executed in Mathematica. Fig. 9A shows the variation of optimal Hex packings along the boundary where $F(\text{Lam}) = F(\text{Hex})$, showing the transformation from circularly IMDS shapes for $\epsilon \lesssim 2$ (and low f) to more polygonal IMDS shape for $\epsilon \gtrsim 2$ (and higher f). This subdomain morphology transition is driven by increasing the cost of packing frustration in the outer corona. This outer corona packing frustration is somewhat relieved by slightly faceting the IMDS, which adopts a rounded hexagonal shape, at the cost of increased interfacial energy and core domain packing frustration. In Fig. 9B, we show the root-mean-square variance of A and B block extension for these equilibrium morphologies along the $F(\text{Lam}) = F(\text{Hex})$ line as function of elastic asymmetry ϵ.

SUPPLEMENTARY NOTE 7. SURFACE SHAPE ANALYSIS

We have developed a set of computational tools to analyze the shape of each IMDS. These tools rely on the construction of the IMDS as a triangulated mesh: an ordered collection of points $\{\mathbf{v}_i\}_{i=1}^{N_v}$, where N_v is the number of vertices, that is grouped into collections of triangular facets $\{f_\mu\}_{\mu=1}^{N_f}$, where N_f is the number of facets.

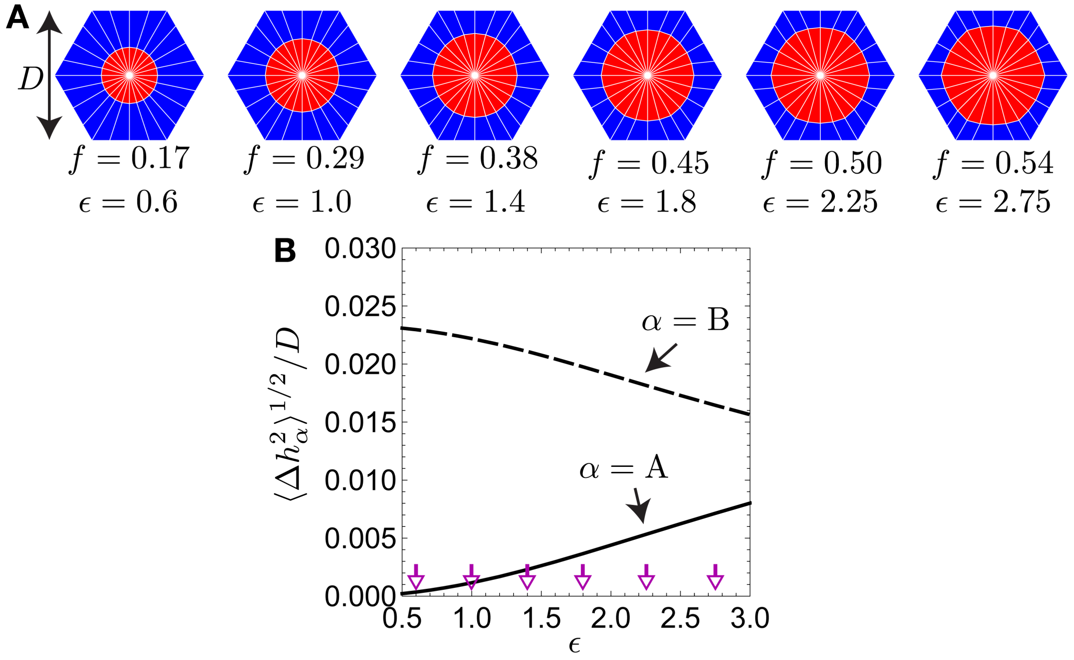

Supplementary Fig. 9. (A) Illustrations of a single Voronoi cell of the Hex phase with IMDS distorted by packing frustration. The number of wedges used to tessellate each cell has been reduced for clarity.(B) Variance in the thickness h_α of block A (solid) and block B (dashed) as a function elastic asymmetry parameter ϵ.

A. IMDS curvature calculation

Smooth surfaces embedded in 3 dimensions are characterized by two types of curvature: Gaussian curvature K_G and mean curvature H. When these surfaces are approximated by meshes, there is no unique definition of the discrete analogues of these curvatures. Instead, there are ways of defining discrete analogues of curvature such that the smooth definitions of curvature are attained in the limit of an infinitely fine mesh [17]. While the generating surfaces are formed from well-refined meshes, after the process of volume balance and joining the wedge dividing planes together, the resulting IMDS does not necessarily have a well-behaved mesh for discrete definitions of curvature; this is especially true for those generated from the skeletal ansatz. Thus, we adopt method of calculating curvatures based fitting smooth surfaces to point clouds, a technique that has proven to produce smooth curvature distributions on complex, potentially poorly meshed surfaces [18, 19].

To start, select a vertex $\mathbf{v}_i$ and a collection of $\mathcal{N}$ nearest points to that vertex, as determined by the Euclidean distance. This neighborhood of $\mathbf{v}_i$ defines a surface patch $\mathcal{P}_i$ of the meshed surface. From the selection of points $\mathbf{v}_m \in \mathcal{P}_i$, where $m = 1, \ldots, \mathcal{N}$, we determine the center of mass $\mathbf{v}_i^* = \mathcal{N}^{-1} \sum_m \mathbf{v}_m$ and inertia tensor $\mathcal{I}_i = \mathcal{N}^{-2} \sum_{m,n} (\mathbf{v}_m - \mathbf{v}_i^*) \otimes (\mathbf{v}_n - \mathbf{v}_i^*)$. We use the eigenvectors of this inertia tensor to construct a local orthonormal basis $\{\hat{\mathbf{e}}_1, \hat{\mathbf{e}}_2, \hat{\mathbf{e}}_3\}$, where we select $\hat{\mathbf{e}}_3$ to align with the minimum eigenvalue of $\mathcal{I}_i$, representing the direction of minimal vertex variation. We represent each point $\mathbf{v}_m \in \mathcal{P}_i$ in the patch via a center of mass coordinate system, $\mathbf{v}_m - \mathbf{v}_i^* = u_m \hat{\mathbf{e}}_1 + v_m \hat{\mathbf{e}}_2 + w_m \hat{\mathbf{e}}_3$.

Next, given the collection of points (u_m, v_m, w_m), we fit a quadric form

$$h(u, v) = a_1 u^2 + a_2 v^2 + a_3 uv + b_1 u + b_2 v + c \qquad \text{(Supplementary Equation 42)}$$

via least squares by minimizing the sum $\sum_m |h(u_m, v_m) - w_m|^2$ over the free parameters a_1, a_2, a_3, b_1, b_2, and c. The result is a smooth approximation $\mathbf{r}_i(u, v) = u\hat{\mathbf{e}}_1 + v\hat{\mathbf{e}}_2 + h(u, v)\hat{\mathbf{e}}_3$ of the surface patch $\mathcal{P}_i$ in the Monge representation [20]. Using the Monge representation, the Gaussian curvature is given by

$$K_G(u, v) = \frac{(\partial_{uu} h)(\partial_{vv} h) - (\partial_{uv} h)^2}{[1 + (\partial_u h)^2 + (\partial_v h)^2]^2} \qquad \text{(Supplementary Equation 43)}$$

and the mean curvature is given by

$$H(u, v) = \frac{[1 + (\partial_v h)^2]\partial_{uu} h + [1 + (\partial_u h)^2]\partial_{vv} h - 2(\partial_u h)(\partial_v h)\partial_{uv} h}{2\,[1 + (\partial_u h)^2 + (\partial_v h)^2]^{3/2}} \,. \qquad \text{(Supplementary Equation 44)}$$

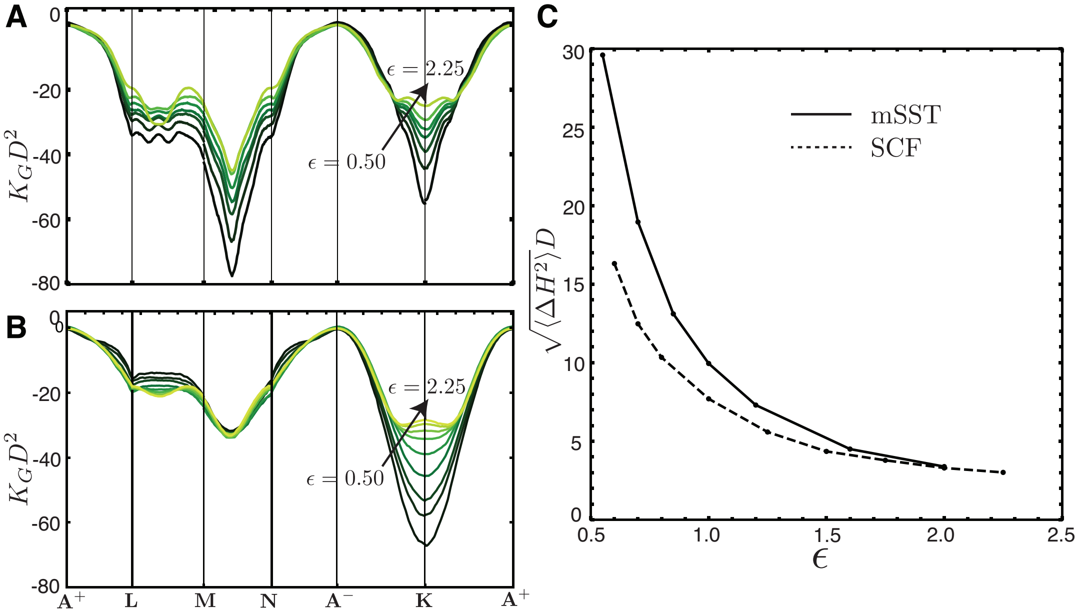

Supplementary Fig. 10. Supplement to Fig. 5. Band diagrams showing the variation of Gaussian curvature along the path defined in Fig. 1D are shown for IMDSs generated by (A) medial SST and (B) SCF. (C) shows the variance in mean curvature as a function of elastic asymmetry ϵ.

Evaluations of $K_G(u, v)$ and $H(u, v)$ yield approximate curvatures for each point $\mathbf{v}_m \in \mathcal{P}_i$: $K_{G,m} = K_G(u_m, v_m)$ and $H_m = H(u_m, v_m)$.

We repeat this process for every point $\mathbf{v}_i$ in the mesh. The collection of surface patches $\bigcup_i \mathcal{P}_i$ yields an atlas that covers the meshed surface. Since every surface patch contains $\mathcal{N} + 1$ points, each of which generates its own surface patch, each surface patch intersects at least $\mathcal{N}$ other surface patches, yielding many measurements of the surface curvature. Define $K_{G,j|i}$ to be the Gaussian curvature of point $\mathbf{v}_j$ as measured in patch $\mathcal{P}_i$; there is a similar definition for mean curvature. We then define an accepted value $\overline{K_{G,j}}$ as the *average* of $K_{G,j|i}$ over all patches $\mathcal{P}_i$ containing $\mathbf{v}_j$; we similarly define the accepted mean curvature $\overline{H}_j$. While the choice of neighborhood size $\mathcal{N}$ yields different resolutions of curvature distribution, we find that for our meshes, $\mathcal{N} = 50$ yields a reasonable trade-off between measuring a smooth curvature distribution that "averages-out" features due to the discreteness of the mesh and nonetheless retaining fine features of the curvature distributions.

1. *Skeletal IMDS curvature*

For the IMDS generated from skeletal SST, we introduce additional steps to smooth the mesh before measuring a curvature distribution. We find this to be necessary since the association map between mesh elements and the gyroid skeleton is not smooth in the vicinity of a node. Proceeding with the patch fitting algorithm discussed above, the mesh undergoes two pre-processing steps. We start by approximating the normal vector at each vertex in the mesh as the average of the normal vectors of the surrounding facets. The angle between the normal vectors of neighboring faces gives an estimate of mesh curvature at the scale of a facet. We identify the roughest regions of the mesh by selecting vertices that have a maximum nearest neighbor normal vector change greater than $\pi/8$. The coordinates of these problem vertices are then averaged with their nearest neighbors. We then identify the longest edges in the mesh, thresholding by the average edge length plus the standard deviation. From this list of edges, we identify vertices with at least 3 long edges and average the coordinates of those vertices with any neighboring vertices that are not in the list of "problem" vertices.

After these pre-processing steps, we use the same patch fitting algorithm for calculating curvature to further smooth the mesh. Since each surface patch has an associated quadratic fit, $h(u, v)$, we can use the quadratic fit for a given patch $\mathcal{P}_i$ to adjust each vertex $\mathbf{v}_j$ to a new position $\tilde{\mathbf{v}}_j$ that lies on the smooth approximation of the surface patch. A given coordinate $\mathbf{v}_j$ exists in multiple surface patches and thus has multiple smooth images; we shall denote the image of $\mathbf{v}_j$ under the map induced by the quadratic fit to patch $\mathcal{P}_i$ as $\tilde{\mathbf{v}}_{j|i}$. Much like the averaging process for

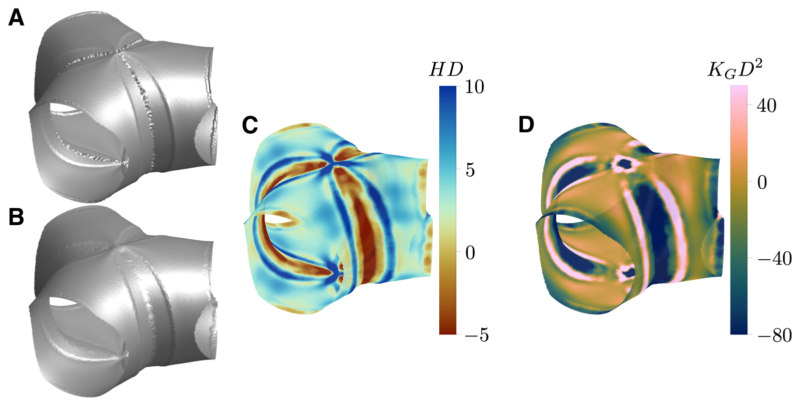

Supplementary Fig. 11. Mesh processing for skeletal IMDS, (A) pre-smoothing and (B) post-smoothing. (C) Mean curvature and (D) Gaussian curvature distributions of the smoothed mesh.

determining surface curvatures, we map each vertex $\mathbf{v}_j$ to the average of $\tilde{\mathbf{v}}_{j|i}$ over all containing patches.

We can iterate the pre-processing and mesh smoothing steps as many times as necessary. Figure 11 shows the result after four iterations of the patch fitting algorithm.

B. "Band" diagrams

In our analysis, we introduce a number of quantities, such as such as domain thickness, curvature, and chain tilt, that vary over a given IMDS. We have found it useful to plot these quantities along a closed path on the IMDS that passes through various symmetry points, much like electronic band structure diagrams. Here, we outline this plotting method.

Let $f(\mathbf{r})$ be a scalar field that takes values at points $\mathbf{r}$ on an IMDS and let $\boldsymbol{\gamma}(s)$ represent a closed path on the surface of the IMDS that passes through symmetry points to be defined below. Here, s is a parameter that varies from 0 to $s_{\max}$ and $\boldsymbol{\gamma}(L) = \boldsymbol{\gamma}(0)$ for a closed loop. The resulting "band" diagram is a plot of f along $\boldsymbol{\gamma}$, namely $f(s) = f \circ \boldsymbol{\gamma}(s)$. While we can, in general, choose any path along the surface, we choose a path that passes through a selection of symmetry points. Each segment of the patch γ can be represented as the intersection of the IMDS with a plane that joins sets of symmetry points:

- $\mathbf{A}^+$ and $\mathbf{A}^-$ lie along [111] and pierce the nodal region, forming an axis of 3-fold rotational symmetry.
- $\mathbf{K}$ is located at a point of 2-fold rotational symmetry, on the saddle-like region between two struts.
- $\mathbf{L}$, $\mathbf{M}$, and $\mathbf{N}$ lie on the plane that bisects a strut. $\mathbf{L}$ and $\mathbf{N}$ are co-planar with $\mathbf{A}^+$ and $\mathbf{A}^-$.
- $\mathbf{M}$ and $\mathbf{K}$ lie on a plane that cuts transversely through the 3-fold symmetry axis.

Therefore, the path $\mathbf{A}^+ \to \mathbf{L} \to \mathbf{M} \to \mathbf{N} \to \mathbf{A}^- \to \mathbf{K} \to \mathbf{A}^+$ can be obtained from the intersection of the IMDS with three cutting planes shown in Fig. 12: (i) the plane formed by the points $\mathbf{A}^+$, $\mathbf{L}$, $\mathbf{N}$, $\mathbf{A}^-$, (ii) the plane formed by the points $\mathbf{L}$, $\mathbf{M}$, $\mathbf{N}$, and (iii) the plane formed by the points $\mathbf{A}^+$, $\mathbf{K}$, $\mathbf{A}^-$.

To create continuous paths from the intersection of each cutting plane with the mesh, we first identify every mesh edge that pierces through a cutting plane. These edges are ordered pairs of vertices, $e_{ij} = (\mathbf{v}_i, \mathbf{v}_j)$, which each form a line $\mathbf{e}_{ij}(\lambda) = (1-\lambda)\mathbf{v}_i + \lambda\mathbf{v}_j$, for $\lambda \in [0,1]$. For each cut edge e_{ij}, we determine the value λ^* that intersects with the plane, using the formula

$$\lambda^*_{ij} = \frac{-(\mathbf{v}_i - \mathbf{p}_{\text{plane}}) \cdot \hat{\mathbf{n}}_{\text{plane}}}{(\mathbf{v}_j - \mathbf{v}_i) \cdot \hat{\mathbf{n}}_{\text{plane}}}\,, \qquad \text{(Supplementary Equation 45)}$$

where $\mathbf{p}_{\text{plane}}$ is a point that lies in the cutting plane and $\hat{\mathbf{n}}_{\text{plane}}$ is the plane's unit normal vector. The list of plane-mesh intersection points $\mathbf{e}_{ij}(\lambda^*_{ij})$ is then ordered by the Euclidean distance of each point from a given end point of the path (for example, we choose $\boldsymbol{\gamma}(0)$ to lie at $\mathbf{A}^+$). This process results in an ordered collection of points $\boldsymbol{\gamma}_i$ that are linear interpolations of mesh vertices.

Most of the data $f(\mathbf{r})$ that we plot are defined on mesh facets. To evaluate on the path, we first find approximate values of f at mesh vertices by averaging over the evaluations of f at facets surrounding a given vertex. We then use

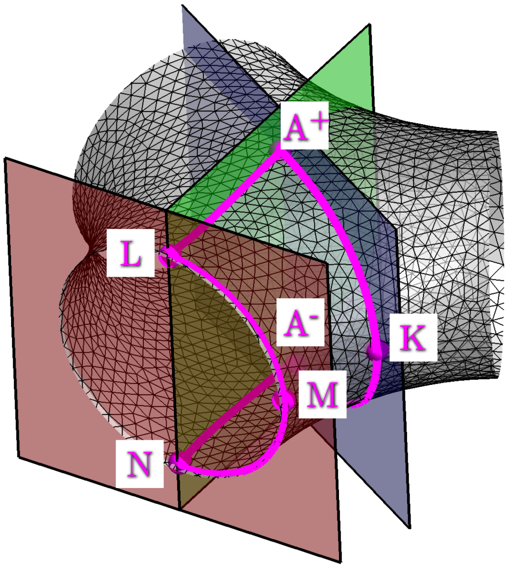

Supplementary Fig. 12. The three slicing planes passing through symmetry points of the DG nodal region used to construct the "band" diagrams used to plot various quantities that vary over the IMDS.

linear interpolation to find values of $f_i = f(\boldsymbol{\gamma}_i)$ that lie along cut edges. The result is an ordered collection of values $(\boldsymbol{\gamma}_i, f_i)$. Finally, we make this path continuous over the entire mesh via linear interpolation.

To standardize the length between each path segment, we choose a "standard" ruler for path lengths. Our standard ruler is the level set $\sin(2\pi x)\cos(2\pi y)+\sin(2\pi y)\cos(2\pi z)+\sin(2\pi z)\cos(2\pi x) = 1$, which has interval lengths $s_{\mathbf{A^+L}} = s_{\mathbf{NA^-}} \simeq 0.177$, $s_{\mathbf{LM}} \simeq 0.193$, $s_{\mathbf{MN}} \simeq 0.183$, and $s_{\mathbf{A^-K}} = s_{\mathbf{KA^+}} \simeq 0.237$.

SUPPLEMENTARY NOTE 8. CHAIN TILT AT THE IMDS

The medial map as defined in eq. Supplementary Equation 13 ensures that the normal $\hat{\mathbf{N}}_{\rm G}$ to a triangular facet belonging to $\mathbf{G}$ is parallel to the centroidal vector $\mathbf{C}$. However, the normal $\hat{\mathbf{N}}_{\rm I}$ of the dividing plane at a given wedge is not generally collinear with $\mathbf{C}$ but makes an angle θ where $\hat{\mathbf{N}}_{\mathbf{I}} \cdot \hat{\mathbf{C}} = \cos\theta$. Within medial SST, we assume that the chain extension is along straight lines connecting the centroids of the faces on the IMDS to those on the terminal boundary $\mathbf{T}_\alpha$.

To test the validity of medial packing we compare the IMDS tilt pattern from medial SST to SCF calculations at finite $\chi N (= 75)$. We compute SCF solutions for DG from the PSCF code [21] and use these solutions to compute both the shape of the IMDS as well as the vector order parameter $\mathbf{p}(\mathbf{x})$ that points along the mean field trajectory of chains using the methods described in [22]. Briefly, this order parameter derives from derivatives of the segment distribution functions $q(n, \mathbf{x})$ and $q^\dagger(n, \mathbf{x})$ which describe the statistical weight of chain segments extending from their free ends at their A and B blocks, respectively, to the $n^{\rm th}$ segment at point $\mathbf{x}$. Given mean-field solutions for these distribution functions, the segment polar order parameter for the $\alpha^{\rm th}$ component is given by

$$\mathbf{p}_\alpha(\mathbf{x}) = -\frac{V a_\alpha}{N}\int_{n\in\alpha} {\rm d}n\, \Big[q^\dagger \nabla q - q\nabla q^\dagger\Big], \qquad \text{(Supplementary Equation 46)}$$

where we have directed the orientation from the B to the A ends, to match to convention of the medial SST trajectories. We extract the end distributions from converged solutions of PSCF and compute these derivatives numerically, arriving at the polar order parameter profile of both blocks, from which we compute the segment averaged orientation $\mathbf{p}(\mathbf{x}) = \mathbf{p}_{\rm A}(\mathbf{x}) + \mathbf{p}_{\rm B}(\mathbf{x})$ which can be converted to a unit vector to map the mean direction of chain trajectories, $\hat{\mathbf{p}}(\mathbf{x})$. As in the previous section, we extract the IMDS from the level set condition $\phi_{\rm A}(\mathbf{x}) = 1/2$ and its local normal at these points by $\mathbf{N}(\mathbf{x}) = \nabla\phi_{\rm A}/|\nabla\phi_{\rm A}|$. From these two fields, we compute and map the local tilt profile from $\cos\theta(\mathbf{x}) = \hat{\mathbf{p}}(\mathbf{x}) \cdot \mathbf{N}(\mathbf{x})$ at the IMDS.

In Fig. 13 we color the volume balanced IMDS with the local tilt θ within each wedge, here shown for case of $f = 0.3$ and $\epsilon = 1$ for the free energy medial SST packing. We also compare this result to the case of finite $\chi N = 75$ from SCF in the same figure. The patterns of non-zero tilt implies that the medial sets of $\mathbf{G}$ are not the medial sets

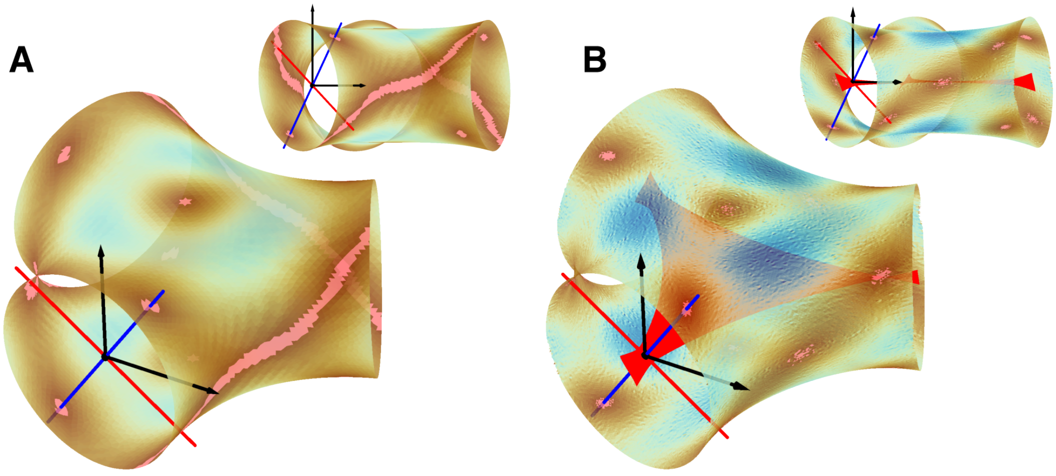

Supplementary Fig. 13. Map of chain tilt θ on the IMDS from (A) SCF and (B) medial SST. Regions of near-normal chain orientations $0 \le \theta \le 1^\circ$ are highlighted in pink. Red and blue line segments show the $70.5^\circ/2$ twist of the IMDS between neighboring nodal regions, oriented tangent and normal to the medial set, respectively. The passage of the medial tangent and normal through locations of near-normal chain orientations is clarified in the rotated view shown in insets.

of the IMDS, and hence chain trajectories are not strictly medial lines. However, there are regions where this tilt is minimal and approaches 0°, where the chain extension is locally medial. These "near-normal" regions are highlighted in pink in Fig. 13 and are remarkably similar in pattern between SCF and medial SST. In particular, half-way along the strut joining the highlighted nodal region to a neighboring region, we find that these low-tilt regions are rotated relative to the nodal axis (the axis threading points $\mathbf{A}^+$ and $\mathbf{A}^-$, the [111] direction), which is consistent with the fact that 3-fold symmetry through this axis. Likewise, the tilt vanishes at point $\mathbf{K}$ which is the location of a 2-fold axis, whose symmetry also requires vanishing of tilt at the IMDS. Additionally, the satellite spot highlighted midway along the strut whose angular location (rotated by $70.5^\circ/2$ relative to the plane of the strut) indicates the coincidence of the IMDS normal and the normal to the underlying medial web, which is the location of another 2-fold axis midway between junctions (between $\mathbf{L}$ and $\mathbf{M}$). Points of medial or near-medial packing are also distributed in a non-trivial pattern away from these symmetry points. Most notably, we observe a low-tilt "scar" shown in both structures that intersects with the strut-bisecting plane (the **LMN**-plane, as defined in our band diagrams) and corresponds to trajectories that terminate at the *edge* of the terminal web. We take the high correspondence between the tilt-patterns predicted by medial SST and SCF (particularly at off-symmetry locations) as a strong evidence that our medial construction accurately captures the underlying and non-trivial patterns of chain packing in finite segregation DG morphologies.

SUPPLEMENTARY NOTE 9. END DISTRIBUTIONS COMPARISONS

Here, we describe the comparison of end distributions for DG structures between medial and skeletal SST calculations and SCF at finite $\chi N = 75$. In particular, we focus on the distribution of A block ends $\rho_{\rm A}(\mathbf{x})$ and the extend to which it concentrates on, or spreads away from, the skeletal graphs in the tubular domains. For SCF the end distribution is directly proportional to the value of the segment distribution function propagating from the free B end along the full length of the chain to the A end at $n = 0$, i.e. $\rho_{\rm A}(\mathbf{x}) \propto q^\dagger(n = 0, \mathbf{x})$, a quantity which can be directly extracted from PSCF solutions.

The free end distributions for SST come from parabolic brush theory (PBT) [2]. PBT only strictly holds for converging (or concave) brushes, i.e. the brushes where chain ends converge towards a central point in space, which is sufficient for our analysis of $\rho_{\rm A}(\mathbf{x})$ in the tubular domains. Converging brushes are defined by an area distribution $A(\tilde{z}) = A_0 \mathcal{A}(\tilde{z})$ that decreases monotonically as a function of the height $\tilde{z}$ above the grafting interface of the brush. Here, A_0 is the area element of the grafting interface (the IMDS) and $\mathcal{A}(\tilde{z})$ is a dimensionless function that has a quadratic form $\mathcal{A}(\tilde{z}) \equiv 1+2\tilde{H}\tilde{z}+\tilde{K}\tilde{z}^2$, per Steiner's law [15]. For smooth surfaces, $\tilde{H}$ is identified as the mean curvature H and $\tilde{K}$ is identified as the Gaussian curvature; for the wedges involved in the medial construction presented here, $\tilde{H}$ and $\tilde{K}$ are the linear and quadratic parts of the area function. For a brush of total height h, PBT predicts a free

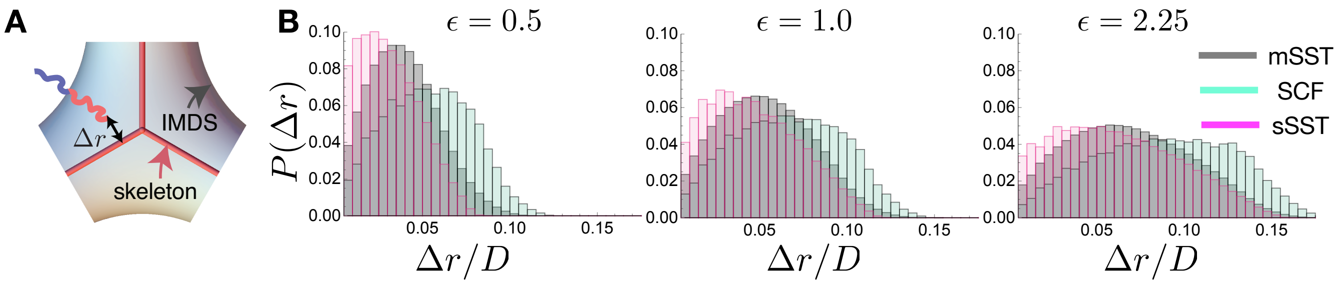

Supplementary Fig. 14. (A) Schematic illustrating the distance of end segments spread away from the skeletal graph and in (B) we show distributions of how end segments are spread away from skeletal graph among medial SST (mSST), skeletal SST (sSST) for f along $F(\text{Lam}) = \text{F}(\text{Hex})$ phase boundary and similarly for SCF.

end probability distribution [1, 2] given by

$$g(\tilde{z}) = \frac{1}{h\mathcal{V}(h)} \left\{ \mathcal{A}(h) \frac{\tilde{z}/h_{\text{A}}}{\sqrt{1-(\tilde{z}/h)^2}} + 2\tilde{H}\tilde{z} \log \frac{\tilde{z}/h}{1+\sqrt{1-(\tilde{z}/h)^2}} - 2\tilde{K}h\tilde{z}\sqrt{1-(\tilde{z}/h)^2} \right\} , \quad \text{(Supplementary Equation 47)}$$

where $\mathcal{V}(h) \equiv 1 + \tilde{H}h + \tilde{K}h^2/3$ is a dimensionless measure of curved brush volume.

In order to quantify the end distributions of A block chains, we set $\tilde{z} = z - h_{\text{d}}$ as the coordinate measuring height with respect to the IMDS. The area function of eq. (Supplementary Equation 22) is then re-cast in this shifted coordinate, prompting new values for A_0, $\tilde{H}$, and $\tilde{K}$:

$$\begin{aligned} A_{\mu,0} &\mapsto A'_{\mu,0} = A_{\mu,0}(1 + 2\tilde{H}h_{\text{d}} + \tilde{K}h_{\text{d}}^2) \\ \tilde{H} &\mapsto \tilde{H}' = \frac{\tilde{H} + \tilde{K}h_{\text{d}}}{\mathcal{A}(h_{\text{d}})} \\ \tilde{K} &\mapsto \tilde{K}' = \frac{\tilde{K}}{\mathcal{A}(h_{\text{d}})} \end{aligned} \quad \text{(Supplementary Equation 48)}$$

Note that the linear end distribution in the form of eq. (Supplementary Equation 47) is normalized such that $\int_0^h \mathrm{d}z\, g(z) = 1$ and is related to the spatial end distribution by additional factors relating to the number of chains per wedge and local area.

Using the fact that the number of chains within wedge μ is $\rho V_\mu(h)/N$ and the local area element at z is $A_\mu(z)$ we have

$$\rho_{\text{A}}(\tilde{z}) = \frac{\rho V_\mu(h)}{N} \frac{g(\tilde{z})}{A'_{\mu,0}\mathcal{A}'(\tilde{z})} . \quad \text{(Supplementary Equation 49)}$$

To compare how concentrated A block ends are within the tubular domains, we convert end distributions to the probability density $P(\Delta r)$ of chain ends at Δr away form the skeletal graph (i.e. each location a chain end $\mathbf{x}$ is mapped on to the closest distance to the skeleton). These distributions are normalized such that $\int_0^\infty \mathrm{d}(\Delta r) P(\Delta r) = 1$. Figure 14 compares the distributions for skeletal and medial SST to SCF predictions ($\chi N = 75$) for three different elastic asymmetries, $\epsilon = 0.5, 1, 2.25$ at the compositions where $F(\text{Lam}) = F(\text{Hex})$, $f = 0.137, 0.293, 0.496$ respectively for medial and skeletal SST and for $f = 0.20, 0.32, 0.51$ for SCF at the same set of ϵ values. We note, in general, that free ends are systematically (if only marginally) more concentrated towards the skeletal graph in the skeletal SST relative to the medial SST, consistent with the fact that terminal ends in the former packing are localized along the graph, while in the latter, terminal ends spread out over web-like surface that extends away from the skeleton. We also, note that ends in $\chi N = 75$ SCF results are always systematically skewed to large Δr than SST predictions, presumably a signature entropic corrections at this finite degree of segregation (e.g. chain backfolding in the brush). Altogether all three distributions are shown to shift to large spread from the skeletal graph for increasing ϵ and f values, consistent with the thickening of the tubular domain with increasing composition.

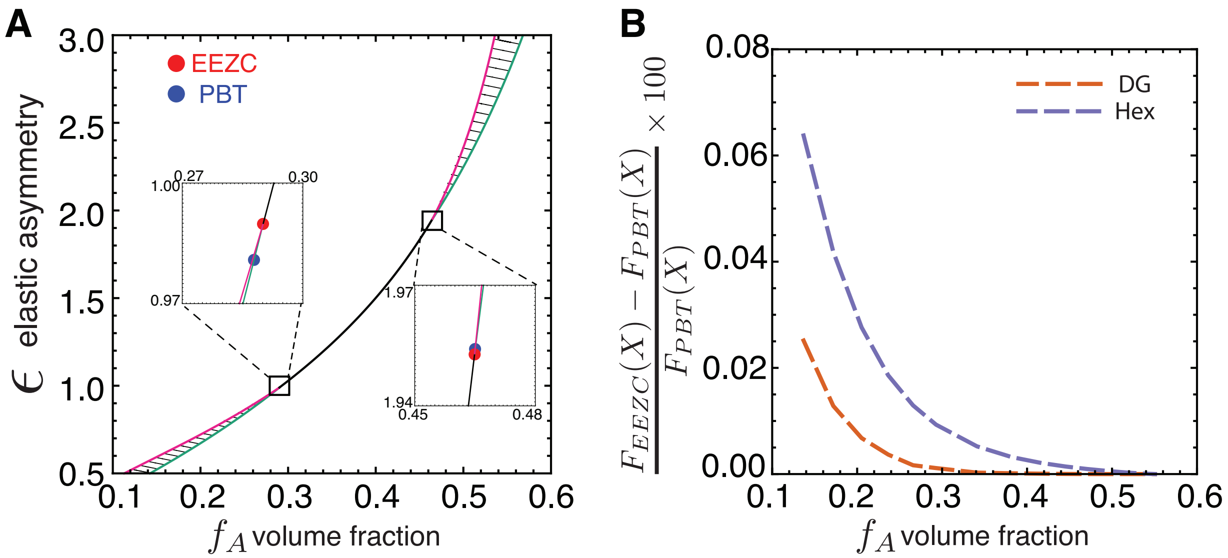

Supplementary Fig. 15. (A) Phase boundaries with end-exclusion zone corrections (EEZC) and both insets highlight triple points with only PBT and with EEZC (B) Relative increase in free energy per chain for DG and Hex are plotted due to EEZC along $F(\text{Lam}) = \text{F(Hex)}$ phase boundary.

SUPPLEMENTARY NOTE 10. END-EXCLUSION ZONE CORRECTED RESULTS

The focus of our presentation relied on the PBT for evaluating the stretching free energy of brush domains, yet it is well known that PBT underestimates the true free energy of convex (outwardly splaying) brushes, as it implicitly allows for a non-positive chain end distribution [1]. The key advantage of the PBT is of course its analytical transparency and geometrically intuitive form eq. (Supplementary Equation 2). Here, we verify that at least for the purposes of modeling the SST thermodynamics of diblocks near to the boundaries between Hex, DG and Lam phase, the PBT is a sufficiently good approximation that corrections due to so-called "end-exclusion zone" (EEZ) effects can be neglected.

We apply the results of a recent formulation of the exact SST of molten brushes accounting for the effects of an EEZ for brushes of arbitrary shape [10]. This approach builds on a previous self-consistent integral formulation [7, 8]. Within an EEZ (close to the anchoring surface at $z = 0$), there are no free chain ends; it is a region where the chain end distribution $g(z)$ evaluates to 0. The presence or absence of an EEZ is controlled by the effect of coefficients $\tilde{H}$ and $\tilde{K}$ in the area distribution $A(z) = A_0(1 + 2\tilde{H}z + \tilde{K}z^2)$; for smooth surfaces, these coefficients are the mean and Gaussian curvatures, respectively. Notably, for concave domains for which $\tilde{H} \leq 0$ it can be shown that the EEZ vanishes, and is only non-zero for convex domains, when $\tilde{H} > 0$, e.g. for the outer brushes of spheres and cylinders, *and* the outer matrix domain of the DG phase. Specifically, the magnitude of the EEZ and its corrections to the PBT are controlled by the ratio of these curvatures to the brush thickness, in this case $\tilde{H}h_{\rm B}$ and $\tilde{K}h_{\rm B}^2$. In ref. [10] is was shown that the predictions of PBT for $H > 0$ are still accurate, with EEZ-corrected free energy increases of order 1% or less appearing for $\tilde{H}h_{\rm B} \lesssim 0.8$. Significant increases on the order of 10% appear for curvatures $\tilde{H}h_{\rm B} \gtrsim 1.6$.

To assess the importance of EEZ-corrections for the convex domains central to this study, we also recalculated the free energies Hex and DG using the exact results available from [10]. From that work we derived $\Delta F(\tilde{H}h_{\rm B}, \tilde{K}h_{\rm B}^2)$, the fractional correction to the PBT stretching free energy due to the EEZ. Given a local facet μ with corresponding values of local curvature coefficients $\tilde{H}h_{\rm B}$ and $\tilde{K}h_{\rm B}^2$ we simply multiply the 2nd moments of the region $I_{\mu,{\rm B}}$ by $\left[1 + \Delta F(\tilde{H}h_{\rm B}, \tilde{K}h_{\rm B}^2)\right]$, and the remainder of the variational calculation for Hex and DG proceeds as described for the PBT.

The change in the phase diagram due to EEZ-corrected stretching free energies is shown in Fig. 15A. At the scale of the original phase diagram, the boundaries between competing Lam, Hex and DG phases are indistinguishable from the PBT case, hence the overall effect of these corrections (which slightly raise $F(\text{Hex})$ and $F(\text{DG})$) on the predicted phase boundaries is minimal. For example, zooming in on the triple points in Fig. 15A, we see the that the EEZ-corrections only displace the lower triple point from the PBT prediction ($f = 0.288, \epsilon = 0.981$) to ($f = 0.290, \epsilon = 0.990$), corrections less than 10^{-2}.

The reason for these very marginal corrections to the phase boundaries is shown in Fig. 15B, where we plot the overall fractional change in the SST free energy for Hex and DG phase along the line where $F(\text{Lam}) = F(\text{Hex})$ (based

on the PBT approximation). Because the outer block stretching contributes ultimately only a (small) part of the total free energy of these structures and the EEZ-corrections to the matrix stretching is modest ($\lesssim 1\%$) for the relevant range of IMDS curvatures, the total change in the free energies due to this correction is very small in comparison to the relevant differences in free energy between these phases as a function of f. However, we note that because the IMDS shapes of DG has negative Gaussian curvature, it is effectively "less convex" than the Hex phase at the corresponding points in composition space, leading to markedly reduced EEZ-corrections to its free energy. Hence, we observe a systematic, if modest, shift of these corrections to slightly increase the window of DG stability relative to Hex.

[1] A. N. Semenov, Contribution to the theory of microphase layering in block-copolymer melts, Journal of Experimental and Theoretical Physics **88**, 1242 (1985).

[2] S. T. Milner, T. A. Witten, and M. E. Cates, Theory of the grafted polymer brush, Macromolecules **21**, 2610 (1988).

[3] J. L. Goveas, S. T. Milner, and W. B. Russel, Corrections to Strong-Stretching Theories, Macromolecules **30**, 5541 (1997).

[4] A. Reddy, X. Feng, E. L. Thomas, and G. M. Grason, Block copolymers beneath the surface: Measuring and modeling complex morphology at the subdomain scale, Macromolecules **54**, 9223 (2021).

[5] P. D. Olmsted and S. T. Milner, Strong Segregation Theory of Bicontinuous Phases in Block Copolymers, Macromolecules **31**, 4011 (1998).

[6] A. E. Likhtman and A. N. Semenov, Stability of the OBDD Structure for Diblock Copolymer Melts in the Strong Segregation Limit, Macromolecules **27**, 3103 (1994).

[7] R. C. Ball, J. F. Marko, S. T. Milner, and T. A. Witten, Polymers grafted to a convex surface, Macromolecules **24**, 693 (1991).

[8] V. A. Belyi, Exclusion zone of convex brushes in the strong-stretching limit, The Journal of Chemical Physics **121**, 6547 (2004).

[9] M. W. Matsen, The standard Gaussian model for block copolymer melts, Journal of Physics: Condensed Matter **14**, R21 (2002).

[10] M. S. Dimitriyev and G. M. Grason, End exclusion zones in strongly stretched, molten polymer brushes of arbitrary shape, J. Chem. Phys. **155**, 224901 (2021).

[11] S. T. Milner, Chain Architecture and Asymmetry in Copolymer Microphases, Macromolecules **27**, 2333 (1994).

[12] The CGAL Project, *CGAL User and Reference Manual*, 5th ed. (CGAL Editorial Board, 2021).

[13] G. E. Schröder, S. J. Ramsden, A. G. Christy, and S. T. Hyde, Medial surfaces of hyperbolic structures, The European Physical Journal B - Condensed Matter **35**, 551 (2003).

[14] C. H. Rycroft, Voro++: A three-dimensional voronoi cell library in c++, Chaos: An Interdisciplinary Journal of Nonlinear Science **19**, 041111 (2009), https://doi.org/10.1063/1.3215722.

[15] S. Hyde, B. W. Ninham, S. Andersson, K. Larsson, T. Landh, Z. Blum, and S. Lidin, Chapter 4 - beyond flatland: The geometric forms due to self-assembly, in *The Language of Shape*, edited by S. Hyde, B. W. Ninham, S. Andersson, K. Larsson, T. Landh, Z. Blum, and S. Lidin (Elsevier Science B.V., Amsterdam, 1997) pp. 141–197.

[16] G. M. Grason and R. D. Kamien, Interfaces in diblocks: A study of miktoarm star copolymers, Macromolecules **37**, 7371 (2004), arXiv:0404409 [cond-mat].

[17] K. Crane and M. Wardetzky, A glimpse into discrete differential geometry, Notices of the American Mathematical Society **64**, 1153 (2017).

[18] V. Krishnamurthy and M. Levoy, Fitting smooth surfaces to dense polygon meshes, in *Proceedings of the 23rd annual conference on Computer graphics and interactive techniques* (1996) pp. 313–324.

[19] I. K. Douros and B. Buxton, Three-dimensional surface curvature estimation using quadric surface patches (2002).

[20] R. D. Kamien, The geometry of soft materials: a primer, Rev. Mod. Phys. **74**, 953 (2002).

[21] A. Arora, J. Qin, D. C. Morse, K. T. Delaney, G. H. Fredrickson, F. S. Bates, and K. D. Dorfman, Broadly Accessible Self-Consistent Field Theory for Block Polymer Materials Discovery, Macromolecules **49**, 4675 (2016).

[22] I. Prasad, Y. Seo, L. M. Hall, and G. M. Grason, Intradomain Textures in Block Copolymers: Multizone Alignment and Biaxiality, Physical Review Letters **118**, 247801 (2017), arXiv:1612.07994.
